# CRTC3, a sensor and key regulator for melanogenesis, as a tunable therapeutic target for pigmentary disorders

**DOI:** 10.7150/thno.66378

**Published:** 2021-10-17

**Authors:** Hanju Yoo, Ha-Ri Lee, Ki-Hyun Kim, Min-Ah Kim, Seunghyun Bang, Young-Ho Kang, Woo-hyung Kim, Youngsup Song, Sung Eun Chang

**Affiliations:** 1 Department of Dermatology, University of Ulsan College of Medicine, Seoul, 05505, Korea.; 2 Department of Biomedical Sciences, University of Ulsan College of Medicine, Seoul, 05505, Korea.; 3 Asan Institute for Life Sciences, Asan Medical Center, Seoul 05505, Korea.

**Keywords:** cAMP- or UV-stimulated melanogenesis, CRTC3/CREB, MITF, melanocytes

## Abstract

**Background:** Although CREB phosphorylation is known to be essential in UVB/cAMP-stimulated melanogenesis, CREB null mice did not show identifiable pigmentation phenotypes. Here, we show that CREB-regulated transcription co-activator 3 (CRTC3) quantitatively regulates and orchestrates melanogenesis by directly targeting microphthalmia-associated transcription factor (MITF) and regulating the expression of most key melanogenesis-related genes.

**Methods:** We analyzed CRTC3-null, KRT14-SCF transgenic, and their crossover mice. The molecular basis of CRTC3 effects on pigmentation was investigated by histology, melanin/tyrosinase assay, immunoblotting, shRNA, promoter assay, qRT-PCR, and subcellular localization. These analyses were carried out in primary cultured melanocytes, mouse cell lines, normal human cells, co-cultures, and *ex vivo* human skin. CRTC/CREB activity screening was performed to identify candidate agents for the regulation of melanogenesis.

**Results:** The coat and skin color of CRTC3-null mice was paler due to a reduction in melanin deposition. Melanogenesis-related genes were reduced in CRTC3-deficient cultured melanocytes and tail skin of CRTC3-null mice. Notably, basal levels of MITF present in CRTC3-null mice were sufficient for melanocytic differentiation/survival. Thus CRTC3-null mice showed a comparable number of epidermal melanocytes compared to control mice. Stem cell factor (SCF) introduction by crossing with KRT14-SCF mice increased epidermal melanocytes and melanin deposition in control and CRTC3-null mice, but the skin color remained still light on the CRTC3-null background. Furthermore, we identified the therapeutic potential of altiratinib to inhibit melanogenesis in human melanocytes and human skin effectively and safely.

**Conclusion:** CRTC3 appears to be a key sensor for melanogenesis and can be used as a reversible and tunable tool for selectively regulating melanogenesis without affecting melanocyte integrity. Thus, CRTC3 can also serve as a screening tool for the discovery of ideal melanogenesis-modulating small molecules.

## Introduction

Most animal species have developed unique skin pigmentation patterns, which are utilized for communicating between individuals within a species, express sexual attractiveness to a potential mate, and most importantly, defend themselves and maintain order within the ecosystem. The skin pigmentation pattern of lower vertebrates can change rapidly through aggregation and dispersion and can match skin patterns with the environmental background, providing protection from predators. Similar but distinct from camouflaging mechanism, the human pigmentation system has also evolved for protection. Ultraviolet radiation (UVR) is a high-threat factor for living organisms, damaging cellular components directly or indirectly by generating reactive oxygen/nitrogen species. Skin tissue located in the outermost layers of the body is directly exposed and highly vulnerable to UV. Melanin is a heterogeneous polymer characterized by high absorbing power for broad-spectrum radiation and reactive species produced by skin tissue. It provides an endogenous self-defense mechanism against UV-induced genotoxic damage and the subsequent malignant transformation 1, 2. However, excessive melanin prevents vitamin D3 synthesis and threatens skin homeostasis. Therefore, melanogenesis must be strictly and reversibly controlled on demand.

Melanin deposition in human skin is tightly regulated by two major steps: the expression of melanin synthesis genes and ensuing enzymatic reactions related to melanin biosynthesis induced by these genes. Microphthalmia-associated transcription factor (MITF) is the master regulator of melanogenesis that integrates various upstream signals and regulates the expression of downstream target genes, including tyrosinase, tyrosinase-related protein-1 (Tyrp-1), and dopachrome tautomerase (DCT) 3. Tyrosinase in the melanosomes catalyzes L-tyrosine hydroxylation to L-dihydroxydroxyphenylalnine (L-DOPA) and the subsequent oxidation of DOPA to DOPA-quinone, thereby providing precursors for melanin and controlling the rate of melanin biosynthesis. L-DOPA-quinone is further catalyzed by DCT and Tyrp-1 and is converted to eumelanin. However, under conditions of low or no DCT and Tyrp-1 expression, it reacts with cysteine and becomes pheomelanin. Thus, the biochemical rate of melanin biosynthesis is determined by tyrosinase activity, but tyrosinase, DCT, Tyrp-1 levels and the eumelanin/pheomelanin ratio are ultimately regulated by MITF.

Although multiple signaling pathways and factors have been shown to be involved in the regulation of melanin biosynthesis, cyclic adenosine monophosphate (cAMP) and cAMP response element-binding protein (CREB) appear to play a principal role in the UVB-induced MITF expression and adaptive melanogenesis. CREB is a ubiquitously expressed basic leucine zipper (bZip) transcription factor in the nucleus and occupies the CRE motif of target genes. Its transcriptional activity is regulated by the phosphorylation status of CREB at Ser133 4. Recently, three isoforms of CREB-regulated transcription co-activators (CRTCs), which are expressed exclusively and/or redundantly in various organs, were identified. It has been reported that alone with CREB phosphorylation, recruitment of CRTCs to the CREB complex is required for the full activation of CREB-mediated transcription 5. Moreover, unlike CREB knockout (KO) mice that died after birth, individual CRTC KO mice survived and exhibited CREB signaling-related traits of appetite, reproduction, glucose, and energy metabolic phenotypes 6-8. Therefore, we hypothesized that genetically engineered CRTC mutant mice might provide an opportunity to elucidate further the physiological role of CREB in skin tissues and of CRTC3 and CREB in UV/cAMP-stimulated melanogenesis.

## Methods

### Animals

All animal studies were conducted according to the protocol approved by the Institutional Animal Care and Use Committee of the Asan Medical Center, Seoul, Korea (2020-02-248). C57BL6/J CRTC3 wild-type (WT) (CRTC3+/+), hetero (CRTC3+/-), and CRTC3-null (CRTC3-/-) mice were reared under temperature-controlled specific pathogen-free conditions with a 12 h light/dark cycle, free access to water, and a normal chow diet (Purina, Pyeongtaek, Republic of Korea). Age-matched experimental animals of both genders, 2-month-old adults and post-natal day 1 pups, were used. C57BL6/J and KRT14-SCF transgene-harbored C57BL6/J mice (KRT14-SCF) were purchased from Jackson Laboratories (Bar Harbor, USA). CRTC3 null mice were made as previously described 8. C57BL6/J WT or CRTC3 null mice were crossed with KRT14-SCF mice, producing KRT14-SCF:C57BL6/J or KRT14-SCF:CRTC3-null, where both mice had the same genetic background as C57BL6/J.

### Cell culture

Mel-Ab normal mouse melanocytes (a gift from Amorepacific Corp., Seoul, Korea) were cultured in Dulbecco's Modified Eagle medium (DMEM, WELGENE, Gyungsan, Korea) supplemented with 10% fetal bovine serum (FBS), 100 nM 12-o-tetradecanoylphorbol-13-acetate (TPA, Sigma-Aldrich, St. Louis, MO, USA), 1 nM cholera toxin (Cayman Chemicals, Ann Arbor, MI, USA), and 1% antibiotic-antimycotic solution (A. A., Thermo Scientific, Rockford, IL, USA). All cells were maintained in a humid environment at 5% CO_2_. B16F10 murine melanoma cells, normal human keratinocytes (NHK), and HaCaT cells (The Korean Cell Line Bank, Seoul, Korea) were maintained in DMEM containing 10% FBS and 1% A. A. solution. Normal human epidermal melanocytes (NHM) of the moderately pigmented type (Invitrogen, Carlsbad, CA, USA) at passage 3-5 were cultured in medium 254 supplemented with human melanocyte growth supplement (Invitrogen, Carlsbad, CA, USA). Co-culture of NHM and NHK HaCaT cells was performed in M254 and DMEM mixed medium, at a seeding ratio of 2:1. NHM were seeded in a 6-well plate at a density of 6 × 10^5^ cells/well. On the next day, HaCaT cells were added to each well at a density of 3 × 10^5^ cells for the co-culture. For stimulation, forskolin (FSK, [3R-(3α,4aβ,5β,6β,6aα,10α,10aβ,10bα)]-5-(Acetyloxy)-3-ethenyldodecahydro-6,10,10b-trihydroxy-3,4a,7,7,10a-pentamethyl-1H-naphtho[2,1-b]pyran-1-one) (Tocris Bioscience, Bristol, England) was used.

### Subcellular localization of CRTC3 and CRTC3 shRNA transfection

Plasmid constructs encoding the CRTC3-EGFP fusion gene were transfected into B16F10 cells using PEI reagents. After 24 h of transfection, CRTC3-EGFP-transfected B16F10 cells were treated with FSK or TPA, and subcellular localization of CRTC3 was monitored by fluorescence microscopy (Observer.Z1, Carl Zeiss, Oberkochen, Germany). To knock down CRTC3 in Mel-Ab and HaCaT cells, specific short hairpin RNA (shRNAi) for CRTC3 was designed and cloned to a pLKO.1 vector. Lentivirus containing shCRTC3i was infected into Mel-Ab cells, followed by the selection of puromycin-resistant cells. A scrambled RNAi (shUSi)was used as a control. All cloning procedures into AgeI and EcoRI sites were performed according to the standard digestion-ligation protocol. For overexpression of CRTC3 in Mel-Ab cells, the gene was cloned in pCDH-CMV-MCS-EF1-puro vector, which has resistance to puromycin for selecting stably transduced cells. Cloning procedures into EcoRI and NotI sites were performed according to the standard digestion-ligation protocol.

### Antibodies

In this experiment, the following antibodies were used: tyrosinase, Tyrp1, DCT, MITF, SOX10, Pmel, Ki67, and PCNA were obtained from Abcam (Cambridge, UK). Total ERK, phospho-ERK, total CREB, and pCREB were purchased from Cell Signaling Technology (Danvers, MA, USA). Another antibody for tyrosinase (Santa Cruz, Dallas, TX, USA) was also used to confirm bands. Antibody for MLANA was obtained from Cell Marque (Rocklin, CA, USA). α-tubulin (Gentex, Holland, MI, USA) and HSP90 (Santa Cruz) were used as internal loading controls. Secondary antibodies used for western blotting were as follows: goat anti-rabbit IgG- horseradish peroxidase (HRP) (1:5,000), goat anti-mouse IgG-HRP (1:5,000), and mouse anti-goat IgG-HRP (1:5,000).

### Western blotting

Total protein was extracted from Mel-Ab cells, washed once with cold PBS, and lysed in protein lysis buffer (1% SDS in 100 mM Tris and 5 mM EDTA, pH 7.4), followed by incubation at 100 °C for 10 min. Protein concentrations were determined using a Bradford assay kit (Biorad, Hercules, CA, USA). Next, 20 μg of protein samples per lane were separated by 5.5% or 8% SDS-polyacrylamide gel electrophoresis and transferred onto 0.45 μm nitrocellulose membranes (GE Healthcare, Chicago, IL, USA). Following blocking with Tris-buffered saline containing 0.5% Tween 20 and 5% BSA, blots were incubated with the appropriate primary antibodies and, after washing, were further incubated with HRP-conjugated secondary antibodies. Protein bands were visualized by incubation using enhanced chemiluminescence (Thermo Fisher Scientific, Cheshire, UK).

### Cell viability analysis and CRTC3/CREB activity screening

The viability of cultured cells was evaluated using the WST or MTT assay (Ez-Cytox Cell Viability Assay Kit, Dogen-Bio Co., Ltd., Seoul, Korea) after seeding into a 24-well culture plate at a density of 6×10^4^ cells/well. After incubation at 37 °C for 72 h, the medium was replaced with the fresh medium for the indicated time period of treatment. Then, the Ex-Cytox reagent was added to the medium in each well and after 1 h of incubation and the absorbance of each well was measured at 450 nm by a microplate reader. For MTT assay, cells were treated with 0.01-1 µM of altiratinib (Selleckchem, Houston, TX, USA) for 3 days. MTT solution (2.5 µg/mL) was added to the culture medium and incubated for 4 h. MTT staining was extracted with DMSO. A small-molecule screening was conducted using reporter plasmids (pGAS-hEVX1Pr-Luc) with Clinical Compound Library (TargetMol, Wellesley Hills, MA, USA).

### Melanin content and cellular tyrosinase activity assay

Mel-Ab cells were plated onto 6-well culture plates at a density of 3 × 10^5^ in DMEM supplemented with 10% FBS and 1% A. A. (without TPA and cholera toxin) were treated with 10 µM FSK. After treating for 72 h, the cells were dissolved in 550 µL of 1 N NaOH at 100 °C for 30 min, and the amount of melanin in the supernatant was measured using a microplate reader at an optical density of 405 nm normalized to the amount of protein used and reported as the percent change relative to that in the untreated controls. A 1 mL sample of dorsal hair or 25 mm^2^ tail skin prepared from 2-month-old WT and CRTC3-null mice were solubilized in 550 µL of 1 N NaOH at 100 °C for 60 min and similarly measured as the cellular melanin content.

The cellular tyrosinase activity was evaluated by measuring the rate of dopachrome formation of L-DOPA. After 24 h incubation, the cells were treated with 10 μM FSK for 3 days, and then the cells were washed by cold PBS and lysed in phosphate buffer (pH 6.8), containing 1% Triton X-100 with repeated freeze/thaw cycles. The lysates were clarified by centrifugation at 15,000 rpm at 4 °C for 10 min. After quantifying the protein levels of the lysate and adjusting the protein concentrations with lysis buffer, 90 µL of supernatant mixed with 10 µL of 10 mM L-DOPA in tyrosinase lysis buffer was incubated at 37 °C. Cellular tyrosinase activity was measured by reading the absorbance at 475 nm using a microplate reader every 10 min for at least 1 h.

### Isolation and primary culture of melanocytes in neonatal mice

The primary melanocytes from newborn control (C57BL6/J) WT and CRTC3-null mice were euthanized by decapitation. The mice were washed by immersion in 70% ethanol for 10 min, then washed by sterile PBS. A superficial longitudinal dorsal incision with a scalpel was made through the entire skin after removing extremities from the mice using surgical scissors. To separate the epidermis from dermis, whole skins of mice were incubated in 5 mg/mL Dispase II (Sigma-Aldrich, St. Louis, MO, USA) diluted in PBS at 37 °C for 1.5 h, allowing the fully spread tissues to float. The epidermis was gently separated from the dermis and chopped into pieced of approximately 2 mm × 2 mm fragments. Then, the tissues were incubated for 10 min in TrypLE (GIBCO, Grand Island, NY, USA) at 37 °C with gentle shaking. The dissociated cells were collected by a 100 µM pore cell strainer and centrifuged (200 g, 10 min). After removing supernatants, the pellets were resuspended mechanically by repeated pipetting up and down with an MGM4 medium kit (CC-3249, Lonza, Basel, Switzerland) with endothelin-3 (ET-3) added (CC-4510, Lonza). The epidermal cell mixtures in pellets were counted and plated on culture dishes at a density of 1 × 10^5^ cells/cm^2^.

### Immunohistochemistry (IHC), immunofluorescence staining, and melanin index

Skin tissues were fixed overnight in 4% paraformaldehyde in PBS at 4 °C and paraffin-embedded. The tissues were cut into 6 μm-thick multiple sections. Hematoxylin-eosin (H&E) (Vector Laboratories, Burlingame, California, USA) staining was performed. The sections were deparaffinized, rehydrated, and processed with antigen retrieval solution (Vector) and then incubated with indicated antibodies. The next day, after washing 3 times with PBS, biotinylated secondary antibodies were added and incubated at room temperature for 30 min. After washing three times with PBS, they were incubated with avidin-peroxidase (ABC kit; Vector) at room temperature for 1 h and were developed using NovaRed substrate kit (Vector). The samples biopsied from mice skin were fixed in 10% neutral buffered formalin and embedded in paraffin. For *ex vivo* human skin cultures, skin tissue was acquired from patients receiving abdomen reduction surgery with informed consent in accordance with IRB number 2014-0837. Skin tissues were briefly washed with 100% EtOH followed by 70% EtOH, cut into approximately 1 cm^2^ sections, and placed on metal grids in 6-well plates in contact with DMEM containing 5% FBS and 5% penicillin/streptomycin under a humidified environment of 5% CO_2_. Culture medium was replaced daily. For UVB-stimulated melanogenesis, skin tissue was exposed to 75 mJ/cm^2^ UVB (TL20W/12RS UV lamps with an emission peak of 310-315 nm, Philips, Eindhoven, Netherlands). After 24 or 72 h, skin tissues were harvested for immunofluorescence and Fontana-Masson staining with western blotting, respectively. The specimens were cut into 4 μm-thick sections, and serial sections were deparaffinized and rehydrated. For antigen retrieval, the sections were heated in antigen unmasking solution (Vector) using a pressure cooker (Biocare Medical, Pacheco, CA, USA) at 120.5 °C for 30 s and 90 °C for 10 s. Then, they were stained with primary antibodies for CRTC3 (1:100), Pmel (1:100), and MLANA (1:50) at 4 °C overnight. FITC-conjugated anti-mouse (1:500) secondary antibody was used to detect CRTC3 and anti-rabbit Alexa Fluor 546 (1:500) was used to detect Pmel and MLANA at 4 °C for 30 min. Images were acquired using a Zeiss LSM 780 laser scanning confocal microscope (Leica; Wetzlar, Germany).

Melanin pigments were visualized using Fontana-Masson stain (Idlabs, Ontario, Canada) and multiple areas were randomly photographed using a phase-contrast microscope (BX53, Olympus, Tokyo, Japan). The melanin index was determined by measuring the stained area normalized to the total epidermal area using ImageJ 1.52a (National Institute of Health) and expressed as percent change relative to the controls.

### Transmission electron microscopy (TEM)

Mel-Ab cells were treated with FSK for 72 h and prepared for electron microscopic analysis. Briefly, cultured cells were detached and fixed in a mixture of 4% paraformaldehyde and 2% glutaraldehyde, followed by osmium tetroxide for 2 h. Two-month-old CTRL (C57BL6/J) and CRTC3-null mice tail tissues were also fixed in the same manner. Then, the fixed samples were dehydrated with ethanol and embedded in epon-araldite resin. Ultrathin sections were stained with 2% uranyl acetate and lead citrate and then examined by Tecnai 10 TEM (Fei, The Netherlands).

### Quantitative real-time PCR (qRT-PCR)

Total cellular RNA was extracted from the cells using a FavorPrep^TM^ Total RNA Purification Mini Kit, according to the manufacturer's instructions (Favorgen, Ping-Tung, Taiwan). Following isolation, the quantity and quality of the RNA were determined using a NanoDrop® ND-1000 Spectrophotometer (ND-1000, NanoDrop Technologies, Wilmington, DE, USA). Single-stranded cDNA was synthesized from 1 μg of total RNA using a Revert Aid First Strand cDNA Synthesis Kit, according to the manufacturer's instructions (Thermo Scientific, Rockford, IL, USA). qRT-PCR was performed using a LightCycler® 480II machine coupled with SYBR Green chemistry (Roche Applied Science, Penzberg, Germany). Initial denaturation was performed at 95 °C for 5 min, followed by amplification at 95 °C for 10 s, 60 °C for 10 s, and at 72 °C for 10 s for 45 cycles. The cDNA obtained was amplified with the primers listed in Table S1.

### Statistical analysis

Data are represented as the mean ± SEM, and statistical significance was determined by an unpaired Student's t-test using GraphPad Prism software. P < 0.05, P < 0.01, and P < 0.001 are represented as *, **, and ***, respectively, and considered statistically significant.

## Results

### Stimulation of CREB activity by protein kinase A (PKA) but not protein kinase C (PKC)

**Figure 1:**
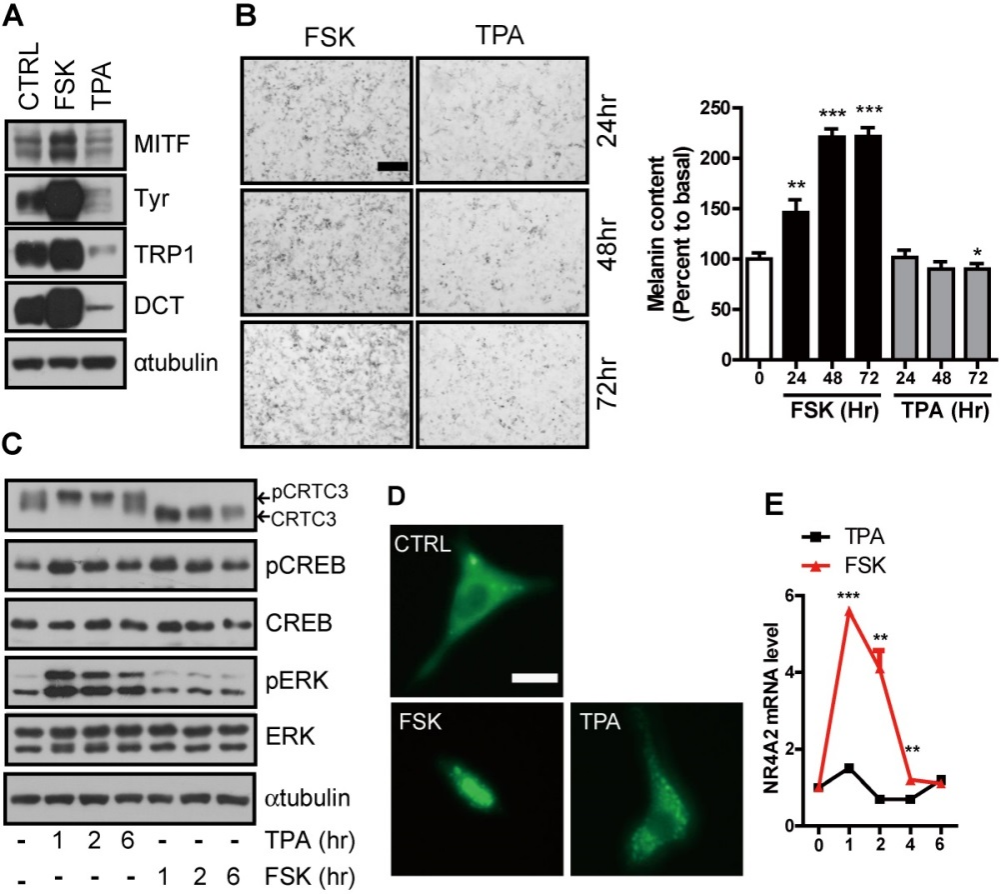
**Stimulation of melanogenesis by cAMP but not PKC signaling pathway. (A)** Western blot analysis of MITF, tyrosinase, Tyrp1, and DCT in Mel-Ab cells treated with FSK or TPA for 72 h. **(B)** Microscopic photographs of the Mel-Ab cells treated with FSK or TPA and melanin content is shown in relative percent to basal melanin content (Bar = 20 µm). **(C)** Western blot analysis of p-CRTC3, CRTC3, CREB, p-CREB, and ERK in Mel-Ab cells treated with FSK or TPA for 1, 2, 6 h. αtubulin was used as a loading control, **(D)** Subcellular localization of CRTC3 (green color) in CRTC3-EGFP-transfected B16F10 cells treated with FSK or TPA (Bar = 200 µm), **(E)** The mRNA expression of NR4A2, a direct target of CREB/CRTC3, by FSK or TPA treatment within 6 h. CTRL: vehicle-treated controls, FSK: forskolin, TPA: 12-o-tetradecanoylphorbol-13-acetate

PKA and PKC are two major signaling pathways that positively correlate with melanocyte proliferation and/or melanin synthesis 9, 10 and induction of Ser^133^ CREB phosphorylation 11. However, unlike PKA, which stimulates both melanocyte proliferation and melanogenesis, PKC activation reduced the number of melanosomes and delayed the onset of melanization 9, 12. Indeed, the expression of genes involved in melanin synthesis and production was increased by FSK, an adenylate cyclase agonist, treatment. However, it was decreased following treatment with the PKC agonist, 12-o-tetradecanoylphorbol-13-acetate (TPA) (Figure 1A-B).

To test whether this signal discrimination by cAMP and PKC is associated with CRTC3, we treated Mel-Ab mouse melanocytes with FSK or TPA and compared their effect on melanocytes. Although CREB expression and phosphorylation induced by FSK and TPA were comparable, CRTC3 phosphorylation was reduced by FSK but dramatically increased by TPA treatment (Figure 1C). Consistent with its phosphorylation pattern, CRTC3 was mainly located in the nucleus in FSK-treated cells while it was enriched in the cytoplasm of cells treated with TPA (Figure 1D). In agreement with the phosphorylation-dependent subcellular localization of CRTC3, the mRNA expression of CREB target genes, such as NR4a2, but not CREB and ERK1/2, was stimulated by FSK treatment with burst-attenuation kinetics but was decreased by TPA (Figure 1E and Figure S1).

### Light hair and skin pigmentation of CRTC3 null mice

**Figure 2:**
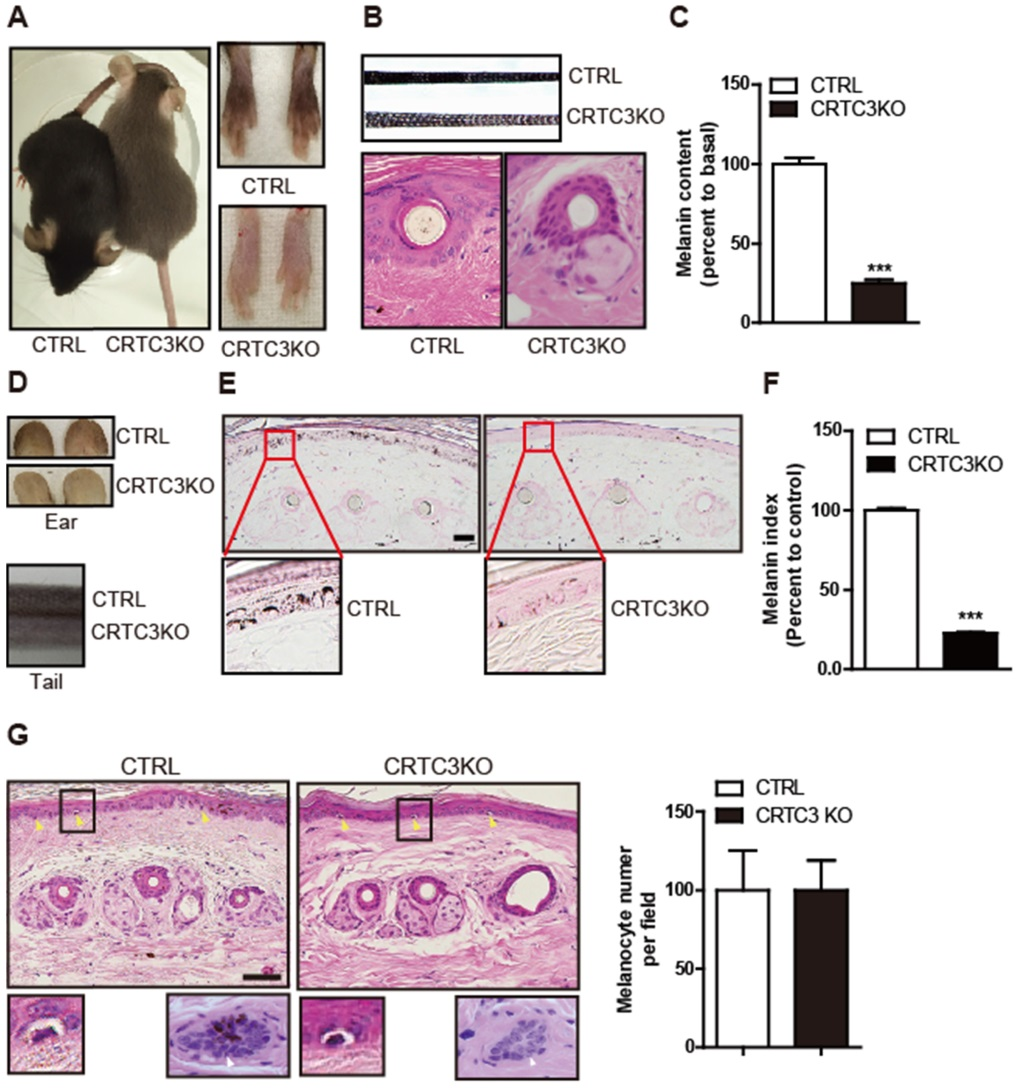
**Light coat color and hypopigmented skin of CRTC3 null mice. (A)** Comparison of coat color between 2-month-old control (WT: CTRL) and CRTC3 null mice. **(B)** Microscopic images of hair and hair follicle and, **(C)** quantification of melanin content in dorsal hair from CTRL and null mice. (n=3 for each group). **(D)** Comparison of the skin color of ear and tail between CTRL and CRTC3 null mice. **(E)** Epidermal melanosomes visualized by Fontana-Masson staining (Bar = 50 µm) and **(F)** melanin index. **(G)** Microscopic images of epidermal melanocytes (yellow arrow), close-up pictures of melanocytes (lower left panel), and hair follicles (melanin deposition) (lower right panel) in tail skin of CTRL and CRTC3 null mice and melanocyte number per field denoted by percent to that of CTRL mice (Bar = 50 µm).

The correlation between CRTC3 and melanogenesis in cultured melanocytes raised the question of whether CRTC3 plays a critical role in skin melanin pigmentation *in vivo*. The coat color of WT and CRTC3 heterozygote mice that were backcrossed 10 times to create a uniform C57BL6/J genetic background was indistinguishable and black, whereas the coat color of CRTC3-null mice was gray (Figure 2A). Phase-contrast microscopy showed that the hair of CRTC3-null mice was less pigmented (Figure 2B). Accordingly, the hair follicular melanin deposition and hair melanin content of CRTC3-null mice were significantly reduced (Figure 2B, C and Figure S2). Importantly, the skin tone of the ear and tail tissues, where melanocytes were reported to be present in the epidermis 13, was also paler in CRTC3-null mice than in the age-matched 2-month-old control mice (Figure 2D). The melanin deposition revealed by Fontana-Masson staining and content in the tail and ear skin tissue of CRTC3-null mice were significantly reduced compared to the WT mice (Figure 2E, F, Figure S3A, and Figure S3B, C).

H&E staining showed that the number of cells surrounded by a transparent halo at the dermal-epidermal junction, presumed to be melanocytes, was comparable in the tail and ear skin tissue of the control and CRTC3-null mice (Figure 2G, Figure S4). Morphologically, no significant abnormalities were found in the gross structure of the hair, follicles, epidermal thickness, or dermal collagen of the RTC3-null mice skin tissue (Figure 2B, G and Figure S3B).

### Downregulation of the melanogenic program in CRTC3 null mice

**Figure 3:**
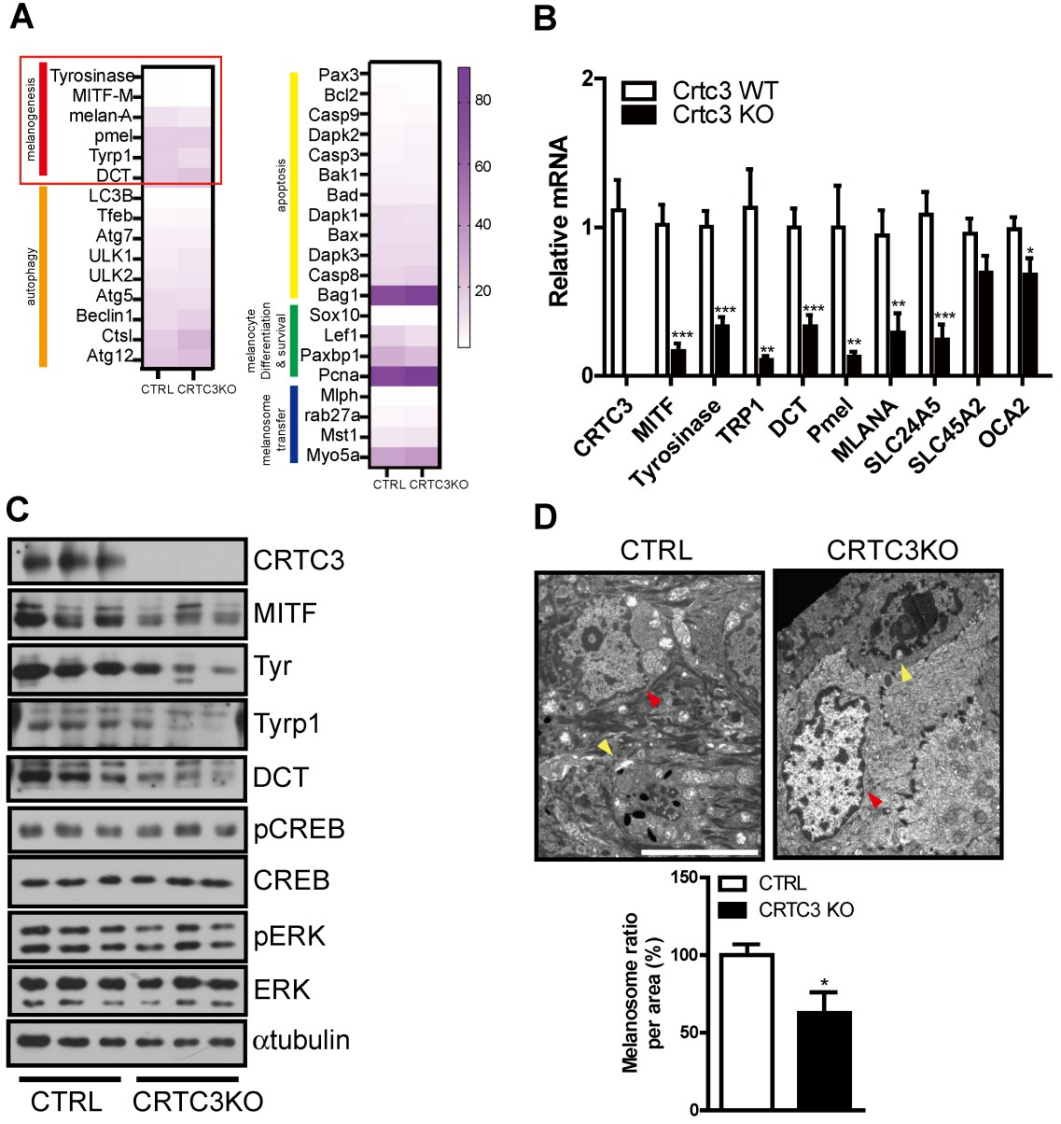
**Downregulation of melanogenic program in CRTC3 null mice. (A)** Heatmap of differentially expressed genes by RNA sequencing experiments in tail skin of 2-month-old CTRL and CRTC3 null mice. **(B)** The mRNA (n=4 for each group) and **(C)** protein levels of melanocyte-relevant genes in tail skin of 2-month-old CTRL and CRTC3 null mice. **(D)** Electron microscopic images of epidermal cells and melanosomes in CTRL and CRTC3 null mice tail tissue. The yellow and red arrowheads indicate melanocyte and keratinocyte, respectively. Melanosome number is expressed ratio per area in CTRL and CRTC3 null mice (Bar = 5 µm).

Since melanin deposition was reduced in the skin of CRTC3-null mice, as shown by Fontana-Masson staining, we focused on the melanin metabolism pathway. First, to understand the pale skin and coat color phenotype of CRTC3-null mice, we conducted RNA sequencing of tail skin tissues isolated from 2-month-old WT and CRTC3-null mice. The expression patterns of numerous genes involved in various pathways, including melanogenesis, melanocyte proliferation/survival, melanin/melanosome degradation, metabolism, and cytokine signaling, were analyzed, and the results are displayed in a heat map and pathway analysis diagram. Static melanin deposition in the skin results from the relative rate of melanin biosynthesis and degradation, and reduced skin melanin deposition is accompanied by downregulation of melanogenesis and/or upregulation of the melanin/melanosome degradation pathway. The transcriptional level of genes involved in melanin/melanosome catabolism, such as autophagy and lysosome, was comparable in WT and CRTC3-null mice. However, a decreased number of gene clusters related to melanogenesis was observed in CRTC3-null mice (Figure 3A). The qRT-PCR and western blot analyses confirmed that mRNA and protein levels of the major melanogenic enzymes, tyrosinase, Tyrp1, and DCT, and their upstream regulator, MITF, were significantly downregulated in CRTC3-null mice (Figure 3B, C). Melanosomal structure and transporter genes were also downregulated in CRTC3-null mice (Figure 3B). Electron microscopy (EM) revealed a lower frequency of melanosomes in tail epidermis of CRTC3-null mice than in control mice (Figure 3D).

### Melanocyte-independent role of CRTC3 in melanogenesis

**Figure 4:**
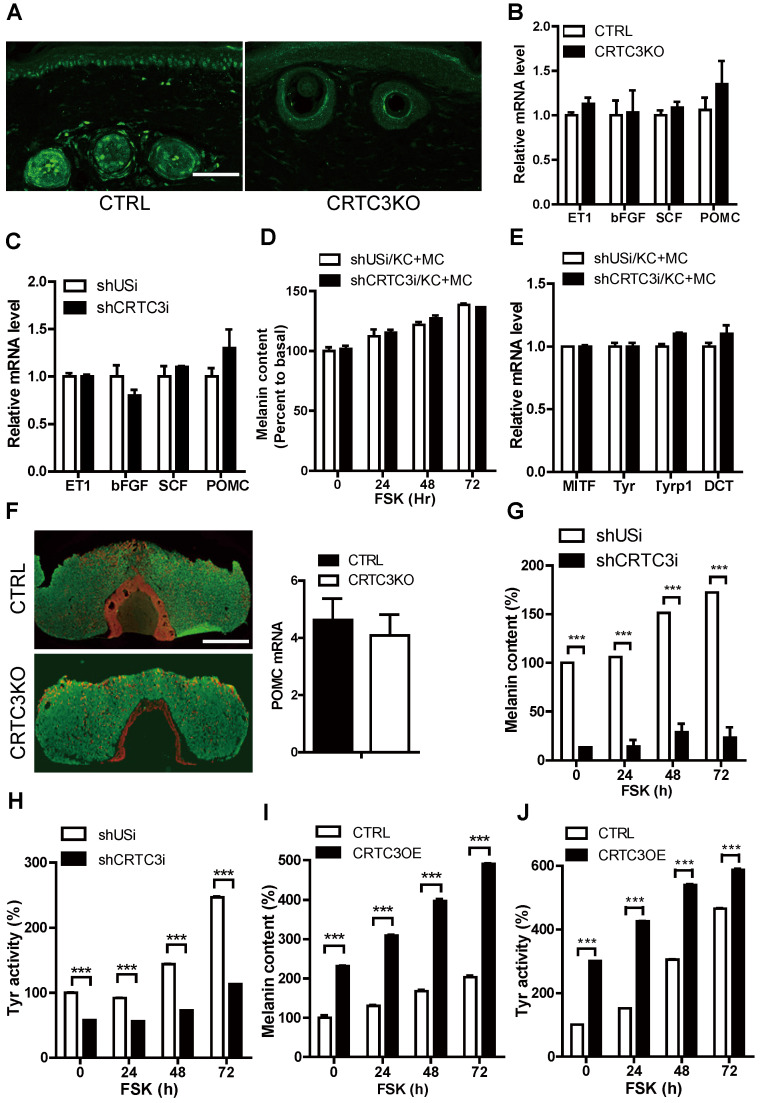
**Melanocyte-independent role of CRTC3 in melanogenesis. (A)** Immunofluorescence staining using CRTC3 antibody (green) with tail skin of control and CRTC3 null mice (Bar = 50 µm). **(B)** mRNA levels of keratinocyte-derived melanogenic paracrine factors in the tail skin of CTRL and CRTC3 null mice and **(C)** in control and CRTC3KD HaCat keratinocytes. **(D)** Melanin content and **(E)** melanogenesis-related mRNA level in Mel-Ab cells co-cultured with either control or CRTC3KD keratinocytes 72 h after with or without FSK treatment. **(F)** Immunofluorescence staining of ACTH (red) and growth hormone (green) (left panel) and mRNA level of POMC (right panel) in the pituitary gland of CTRL and CRTC3 null mice (Bar = 100 µm). **(G)** Melanin content and **(H)** tyrosinase activity in control and CRTC3 knockdown Mel-Ab cells at 0, 24, 48, and 72 h after FSK treatment. **(I)** Melanin content and **(J)** tyrosinase activity in control and CRTC3 overexpressing Mel-Ab cells at 0, 24, 48, and 72 h after FSK treatment.

We performed immunohistochemical analysis using the CRTC3 antibody. As is evident from Figure 4A and Figure S5A, CRTC3 expression was observed in melanocytes and keratinocytes of the mouse skin tissue. We confirmed that CRTC3 was expressed and responded to cAMP signals in the cultured mouse melanocytes and keratinocytes (Figure S5B). Although melanin biosynthesis occurs solely in melanocytes, keratinocytes are the major constituent cells that make up approximately 95% of the epidermal tissues and play pivotal roles in melanogenesis by secreting paracrine factors 14. To test whether the decreased melanogenesis in CRTC3-null mice was caused by the loss of CRTC3 in keratinocytes, we knocked down CRTC3 (CRTC3KD) in HaCaT keratinocytes (Figure S5C) and examined the effect on melanogenesis. The mRNA levels of keratinocyte-derived melanogenic paracrine factors, such as proopiomelanocortin (POMC), endothelin 1 (ET1), stem cell factor (SCF), and bFGF, were comparable in control and CRTC3KD keratinocytes and in the tail skin of WT and CRTC3-null mice (Figure 4B, C). Basal and FSK-stimulated melanin content and mRNA levels of the melanogenic genes of melanocytes co-cultured with CRTC3KD or control keratinocytes were also similar (Figure 4D, E, Figure S5D). Furthermore, the decreased melanogenesis in CRTC3-null mice was likely not caused by systemic effects of the pituitary because POMC mRNA and ACTH peptide levels in the CRTC3-null mice pituitary were also indistinguishable from those observed in the control mice (Figure 4F).

Because of the lack of a significant association between the keratinocyte CRTC3 depletion and melanogenesis, we focused on the melanocyte-independent role of CRTC3 in melanogenesis. We generated CRTC3 KD (Figure S6A) or overexpressing (OE) (Figure S6B) melanocytes and assessed CRTC3 expression levels and melanin biosynthesis. CRTC3 KD cells maintained lower basal melanin content than shUsi-infected control melanocytes and were only partially responsive to FSK-stimulated melanin synthesis and tyrosinase activity (Figure 4G, H and Figure S6C). Conversely, basal melanin content was elevated by ectopic overexpression of CRTC3, whereas FSK treatment increased tyrosinase activity and melanin accumulation in both control and CRTC3-OE cells; however, this increase in tyrosinase activity and melanin accumulation was maintained at a higher level in CRTC3-OE cells than in controls (Figure 4I, J and Figure S6D).

### Regulation of MITF expression by CRTC3 at the transcription level

**Figure 5:**
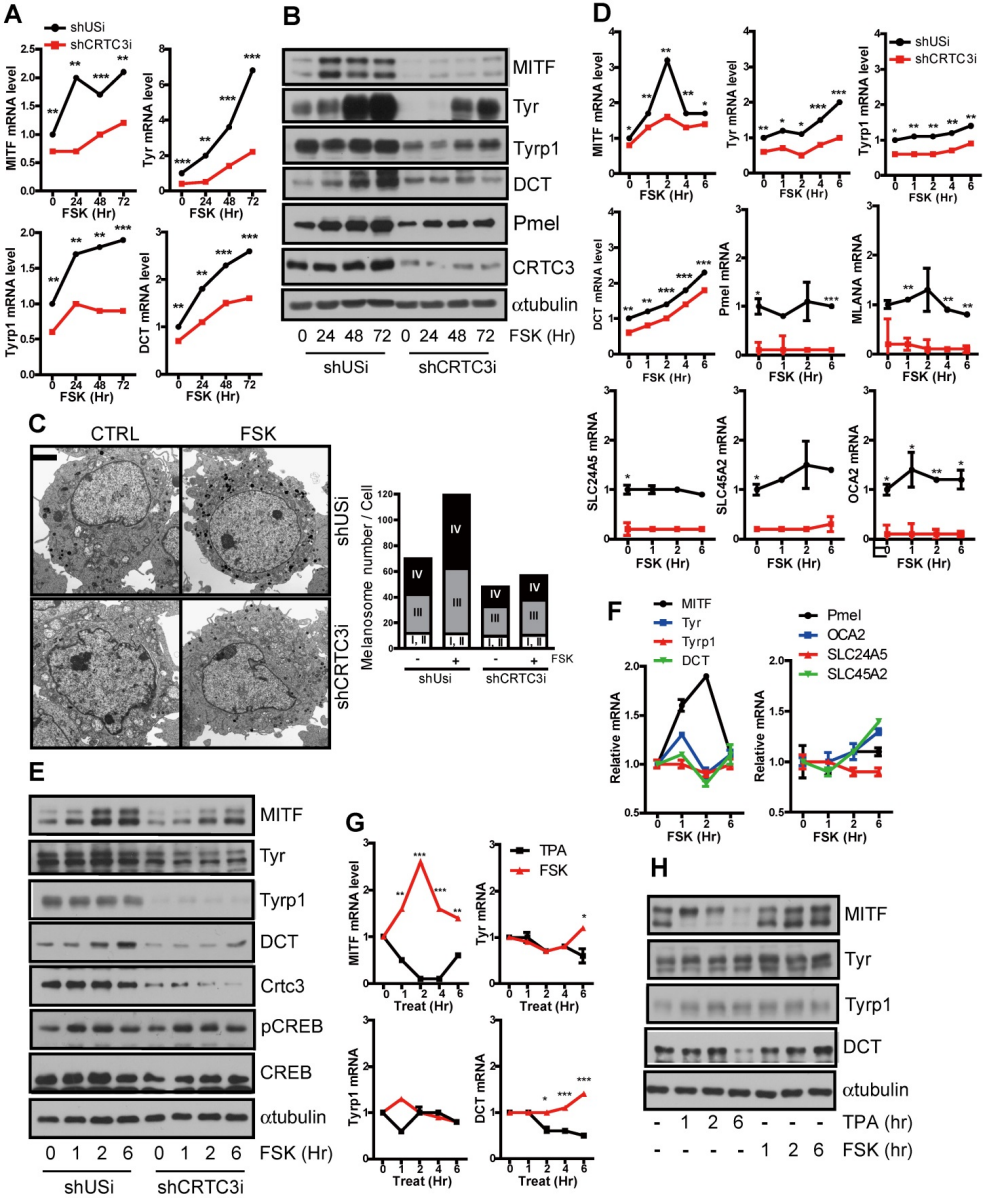
**Decreased melanin synthesis via downregulation of MITF by CRTC3 deficiency. (A)** mRNA and **(B)** protein level of melanogenesis-related genes in control and CRTC3KD Mel-Ab cells at 0, 24, 48, and 72 h after FSK treatment. **(C)** EM images (left panel) and melanosome quantification (right panel) of control and CRTC3KD Mel-Ab cells at 0 and 72 h after FSK treatment (Ba r= 2 μm). **(D)** mRNA and **(E)** protein levels of melanogenesis-related genes in control and CRTC3KD Mel-Ab cells within 6 h after FSK treatment. **(F)** mRNA level of melanogenesis-related genes in primary cultured mouse melanocytes from CTRL mice within 6 h after FSK treatment. **(G)** mRNA and **(H)** protein levels of melanogenesis-related genes in Mel-Ab cells within 6 h after FSK or TPA treatment.

The alteration of cellular tyrosinase activity and resultant melanin content can be explained by the changes in melanogenic enzyme expression levels, such as tyrosinase, Tyrp-1, and DCT 15. Indeed, the basal mRNA and protein expression levels of the melanogenic enzyme, tyrosinase and its upstream regulator, MITF, were upregulated in Mel-Ab cells with CRTC3 overexpression (Figure S7). Conversely, the basal levels of these genes and of the melanosome constituent genes, such as *Pmel*, *MLANA*, and melanosome transporter genes (*SLC24A5*, *SLC45A2*, and *OCA2*), remained low, and the FSK-triggered induction of these genes was markedly attenuated in the CRTC3KD Mel-Ab cells (Figure 5A, B, Figure S8). The degree of melanogenesis evaluated by melanosome maturation was also downregulated in CRTC3KD melanocytes. Although FSK stimulated late stages of melanosomes in control and CRTC3KD Mel-Ab cells, the late-stage melanosome numbers were still lower in CRTC3KD Mel-Ab cells than in the control melanocytes (Figure 5C).

We searched for genes that initiated alteration of melanogenesis in CRTC3-null mice by examining the proximal or direct transcriptional targets of CRTC3 in melanocytes. In agreement with previous reports that the *MITF* gene contains a CRE motif in the promoter region 16, *MITF* transcript level peaked 1-2 h after FSK treatment and returned to the basal level within 6 h; the typical CRTC/CREB target gene expression pattern of *MITF* was mitigated in CRTC3KD cells (Figure 5D). However, any of these melanogenesis-related genes - although their basal level was downregulated in CRTC3KD cells - did not follow cAMP-mediated burst-attenuation kinetics in Mel-Ab (Figure 5D, E) and in B16F10 melanoma cells (Figure S9). Accordingly, MITF promoter activity was sensitive to cAMP stimuli and the CRTC3 expression level (Figure S10). The MITF expression, but not of tyrosinase, Tyrp1, or DCT, was increased within 6 h after FSK treatment in primary melanocytes (Figure 5F). Moreover, MITF transcript and protein levels were increased within 6 h of FSK treatment, supporting an association between CRTC3 activity and MITF expression, and were decreased by TPA treatment (Figure 5G, H and Figure S11).

### Impaired melanogenesis without developmental defects in melanocytes of CRTC3-null mice

**Figure 6:**
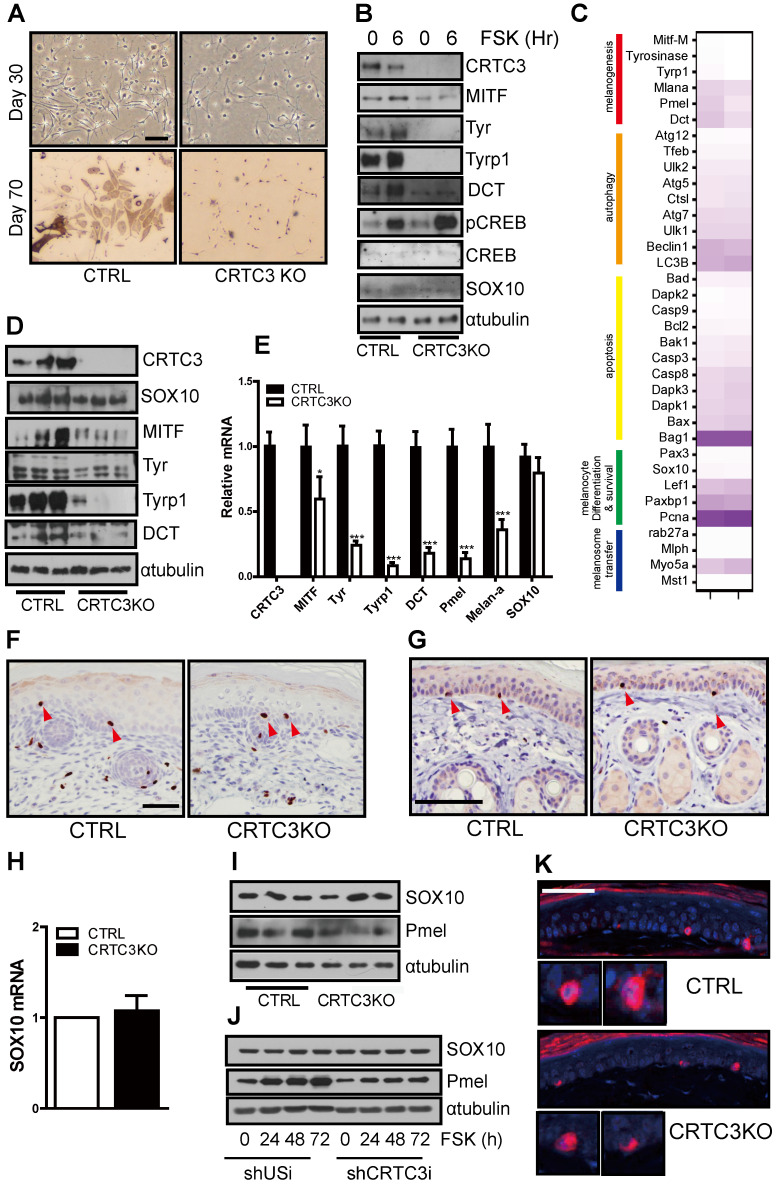
**Downregulation of melanogenesis by the loss of CRTC3 without affecting melanocyte development. (A)** Microscopic images of cultured primary melanocytes from control (CRTL) versus CRTC3 null mice at day 30 and 70 (Bar = 100 µm). **(B)** Protein level of melanocytic genes including CRTC3, MITF, Tyr, Tyrp1, DCT, PCREB, CREB, Pmel, SOX10 in 70-d cultured primary melanocytes from CTRL and CRTC3 null mice in response FSK for 6 h. **(C)** Heatmap of significant differentially expressed melanocyte-relevant genes by RNA sequencing experiments in 1-day-old CTRL and CRTC3 null mice tail skin. **(D)** Protein level of CRTC3, SOX10, MITF, Tyr, Tyrp1, and DCT in CTRL and CRTC3 null mice. α-tubulin was used as an internal loading control. **(E)** Relative mRNA expression of melanogenesis genes, CRTC3 and SOX10 form 1-day-old CTRL and CRTC3 null mice tail skins (n=4, each) as assessed by qRT-PCR. Immunohistochemistry using SOX10 antibodies in the tail skin of **(F)** 1-day-old and **(G)** 2-month-old CTRL and CRTC3 null mice (Bar = 50 µm). **(H)** Relative mRNA level of SOX10 in tail skin of 2-month-old CTRL and CRTC3 null mice. **(I)** Protein levels of SOX10 and Pmel in the tail skin from 2-month-old CTRL and CRTC3 null mice. **(J)** Protein levels of SOX10 and Pmel in control or CRTC3 K/D Mel-ab cells with FSK stimulation for 72 h **(K)** Immunofluorescence staining using Pmel antibody with nuclear DAPI staining of 2-month-old CTRL and CRTC3 null mice tail skin (Bar = 50 µm).

Next, we prepared primary melanocyte cultures from the dorsal epidermis of neonatal mice to confirm whether the decreased melanogenesis in melanocytes was a primary cause of the pigmentary phenotype in CRTC3-null mice. At 30 d from initial isolation and plating of the primary melanocytes, the cellular morphology between WT and CRTC3KO primary melanocytes was similar. However, the number of CRTC3KO primary melanocytes was lower than the control primary melanocytes (Figure 6A), suggesting their slower growth rate than those from the control. After 70 d of culture, the cytoplasmic plumpness and visible melanization in control primary melanocytes were strikingly higher than the CRTC3-null mice primary melanocytes, indicating that melanogenesis was significantly impaired in cultured primary melanocytes derived from CRTC3-null mice (Figure 6A). Western blot analyses showed significant downregulation of the expression of melanogenic genes and a reduction in the expression of MITF, a master regulator of melanogenesis, in CRTC3KO compared to the control (Figure 6B). However, melanocyte development, represented by SOX10 expression, was maintained at a comparable level in CRTC3-null mice, suggesting that epidermal melanocytic differentiation or development was not affected by CRTC3 loss (Figure 6B).

After confirming that CRTC3 was a key regulator of melanogenesis, we determined whether the decreased proliferation rate observed in CRTC3-null mice-derived primary melanocytes affected melanocyte development. Whether the reduced melanin deposition in CRTC3-null mice might be attributable to a decrease in the number of epidermal melanocytes or reduced melanocytic development was examined by comparing the epidermal melanocytes of neonatal and 2-month-old control and CRTC3-null mice. Consistent with the transcriptomic profiles of the neonatal tail skin (Figure 6C), qRT-PCR and immunoblot analysis confirmed that the expression of melanogenic genes, including those encoding the early and late-stage melanogenesis markers, tyrosinase, and Tyrp1, and the melanosome transporter OCA2, was decreased in the neonatal skin of CRTC3-null mice (Figure 6D, E). The epidermal and hair follicular melanin deposition in the tail skin of neonatal CRTC3-null mice was lower than the control mice reflecting the decreased level of melanogenic gene expression. (Figure S12). However, SOX10 levels, representing melanocyte numbers, were comparable in the tail skin of the neonatal control and CRTC3-null mice (Figure 6D, E).

SOX10 expression in skin tissues of post-natal mice was highly restricted in the nerves and neural crest lineage cells, such as melanoblasts and melanocytes 17. Analysis of melanocyte development by determining SOX10 levels in whole skin tissues using qRT-PCR or immunoblotting might lead to a biased assessment. Therefore, we used IHC with various antibodies and evaluated possible developmental issues in melanocytes of CRTC3-null mice. IHC with anti-Ki67 antibody suggested that the proliferation of the epidermal cells, most likely keratinocytes, was not differentially regulated in CRTC3-null mice (Figure S13, S14). As expected, SOX10-positive cells in the tail and dorsal skin of neonatal mice were observed in the hair bulbs and epidermis, and there was no difference in the number of SOX10-stained cells in the epidermis between the control and CRTC3-null mice (Figure 6F and Figure S14). Similarly, the number of SOX10 immuno-positive epidermal melanocytes and SOX10 mRNA and protein levels in the tail skin tissue of 2-month-old CRTC3-null mice were comparable to the levels observed in the control mice (Figure 6G, H, I). SOX10 protein in control and CRTC3KD Mel-Ab cells was also comparable (Figure 6J). Although, basal level of Pmel protein was lower in CRTC3KD Mel-Ab cells and the staining intensity of Pmel appeared to be weaker in CRTC3-null mice, Pmel-positive epidermal melanocyte numbers were comparable between control and CRTC3-null mice (Figure 6K). Regarding the central role of MITF in melanocyte lineage development, the ocular structure of CRTC3-null mice was comparable to the control mice with respect to microphthalmia or decoloration (Figure S15).

### CRTC3 knockout mouse model of humanized skin

**Figure 7:**
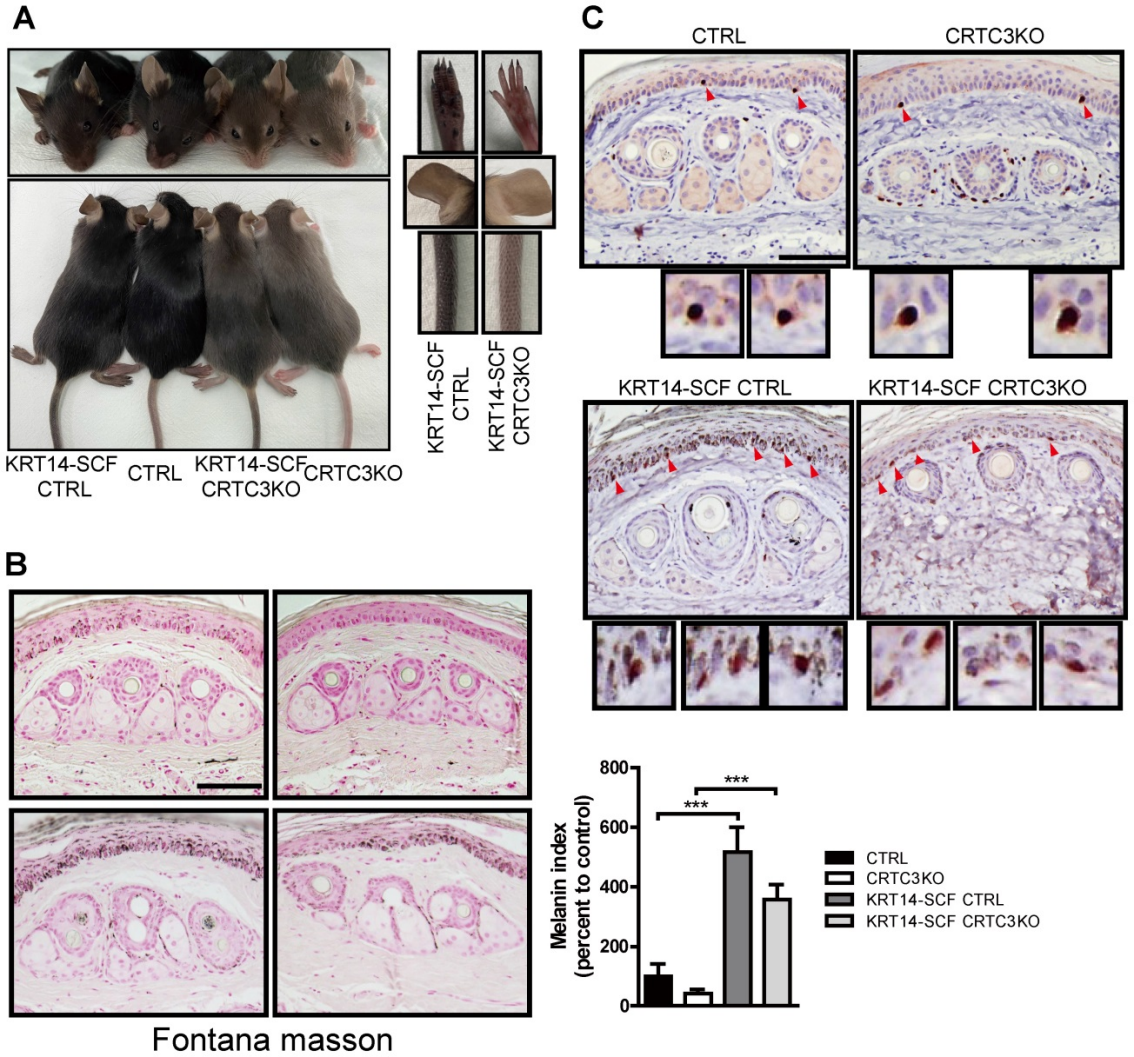
**Light skin color in a CRTC3 null mouse model of humanized skin. (A)** Skin color comparison of CRTC3 null mice crossed with KRT14-SCF transgenic mice compared to CTRL mice crossed with KRT14-SCF transgenic mice, CTRL, and CRTC3 null mice. **(B)** Microscopic images (left panel) and melanin index (right panel) of epidermal melanin deposition assessed by Fontana-Masson staining in tail skin from CTRL, CRTC3 null mice, CTRL mice crossed with KRT14-SCF transgenic mice, and CRTC3 null mice crossed with KRT14-SCF transgenic mice. **(C)** Immunohistochemistry using SOX10 antibodies for melanocytes in the tail skin of CTRL, CRTC3 null mice, control crossed with KRT14-SCF transgenic mice, and CRTC3 null mice crossed with KRT14-SCF transgenic mice. Red arrows indicate SOX10^+^ epidermal melanocytes. Low panels show close-up view of SOX10^+^ melanocytes (Bar = 50 µm).

We employed KRT14-SCF transgenic mice with constitutive expression of the stem cell factor (SCF) by epidermal keratinocytes, resulting in the proliferation and retention of melanocytes in the interfollicular basal layer and pigmentation of the epidermis 18. It is a good model system for investigating epidermal melanocyte biology and the melanocyte response to other melanogenic signals, such as SCF 19. As described previously, compared to C57BL6/J mice, the tail and ear skin color of mice crossed with KRT14-SCF transgenic mice (KRT14-SCF:C57BL6/J) was darker. The KRT14-SCF transgene also induced darker epidermal skin tone in CRTC3-null mice; however, it was not as dark as in the KRT14-SCF transgene-harboring C57BL6/J mice (Figure 7A). The skin color darkeness was in the decreasing order of KRT14-SCF:C57BL6/J> KRT14-SCF:CRTC3-null> C57BL/6J> CRTC3-null mice. Melanosome accumulation in the epidermal tail skin was also observed in the same order (Figure 7B). As expected, the number of epidermal melanocytes, represented by SOX10+ epidermal cells, also increased in control and CRTC3-null mice by KRT14-derived-SCF expression. Importantly, compared to the control KRT14-SCF mice, SOX10+ epidermal melanocyte number in KRT14-SCF CRTC3-null mice was not reduced (Figure 7C), reflecting the exclusive expression of keratin14 in the epidermis, but not in the hair bulb of mice. The coat color of CRTC3-null mice containing the KRT14-SCF transgene was similar to the CRTC3-null mice (Figure 7A).

### Suppression of cAMP- or UVB-induced melanogenesis in human melanocytes and skin culture by altiratinib via CRTC3 phosphorylation

**Figure 8:**
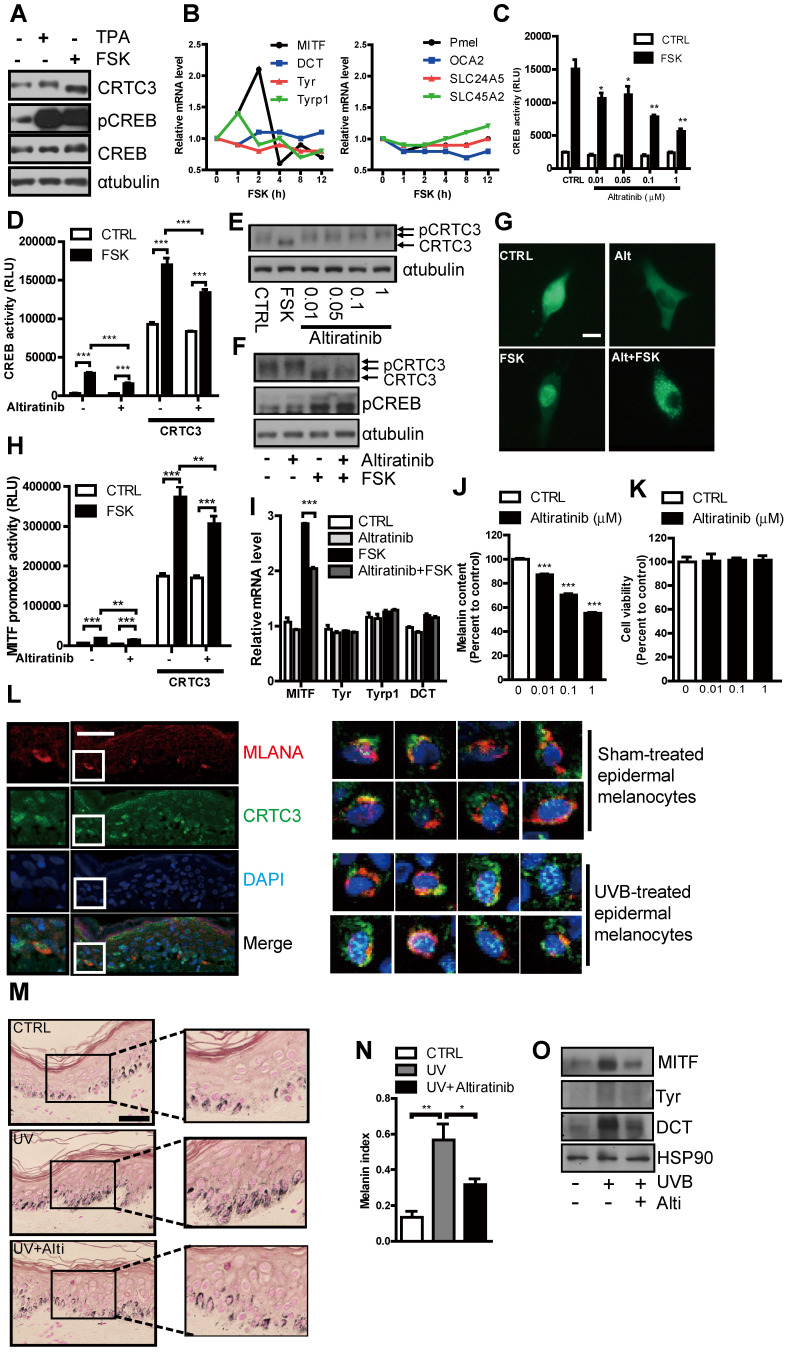
S**uppression of cAMP or UVB-induced melanogenesis in human melanocytes and *ex vivo* human skin culture by altiratinib via CRTC3 phosphorylation. (A)** Western blot analysis of CRTC3 and CREB and melanin content in normal human melanocytes (NHM) treated with FSK or TPA for 1 h. **(B)** mRNA level of melanogenesis-related genes in NHM within 12 h after FSK treatment. **(C)** Altiratinib (0.01-1 μM) dose-dependently suppressed FSK-stimulated transcriptional activity of CREB as measured by hEVX1 promoter activity. **(D)** The effect of 0.1 μM of altiratinib on FSK or CRTC3-stimulated transcriptional activity of CREB. **(E)** Expression levels and phosphorylation status of CRTC3 following 1 h of 0.01-1 μM altiratinib in Mel-Ab mouse melanocyte. **(F)** Expression levels and phosphorylation status of CRTC3 and CREB following 1 h of FSK treatment with or without 1 h of 0.1 μM altiratinib pretreatment. **(G)** Effects of 0.1 μM altiratinib, FSK, and both on the subcellular localization of CRTC3 in B16F10 cells transfected with CRTC3-EGFP (Bar = 100 μm). **(H)** The effect of 0.1 μM of altiratinib on FSK- or CRTC3-stimulated MITF-promoter activity. **(I)** mRNA level in Mel-Ab cells treated 2 h with vehicle (CTRL), 0.1 μM altiratinib, FSK or altiratinib plus FSK. **(J)** Melanin content of NHM treated with altiratinib (0.01-1 μM ) for 72 h. **(K)** The effect of altiratinib (0.01-1 μM) on cell viability of NHM by MTT assay. **(L)** Immunofluorescence staining using MLANA (red), CRTC3 (green) antibody with nuclear DAPI (blue) staining of human skin (Bar = 50 µm) with/without UVB treatment for 24 h. **(M)** Representative images of Fontana-Masson-stained paraffin-embedded sections and **(N)** melanin index, and **(O)** protein expression of MITF, Tyr, and DCT of *ex vivo* human skin exposed to UVB (75 mJ/ cm^2^) with/without 5 μM altiratinib for 96 h (Bar = 50 µm).

Similar to the observations in mouse melanocytes, CRTC3 was expressed in NHM, dephosphorylated in response to the cAMP signal, and specifically regulated MITF transcription with concomitant increase of melanin content. mRNA levels of other melanogenesis-related genes, except MITF, were not elevated in NHM in 12 h after FSK treatment (Figure 8A, B). Having confirmed the central role of CRTC3 for cAMP-mediated melanogenesis in NHM, we performed a small-molecule screening and discovered altiratinib as a candidate small molecule regulating CRTC3/CREB-mediated transcription. In response to cAMP stimulation, altiratinib at 0.01 µM and 0.1 µM concentration suppressed CREB transcriptional activity by 30% and 50%, respectively, with higher concentrations further inhibiting it (Figure 8C). Moreover, altiratinib also attenuated CREB transcriptional activity stimulated by CRTC3 overexpression (Figure 8D). CRTC3 phosphorylation was elevated by altiratinib (Figure 8E) and altiratinib treatment attenuated FSK-stimulated dephosphorylation of CRTC3 without altering CREB phosphorylation (Figure 8F). Consistent with the phosphorylation of CRTC3, altiratinib treatment promoted its cytoplasmic localization and suppressed FSK-stimulated nuclear localization of CRTC3 (Figure 8G). Also, MITF promoter activity stimulated by FSK treatment and/or CRTC3 overexpression was mitigated by altiratinib, and the MITF transcript level induced by 2 h treatment of FSK was significantly decreased (Figure 8H, I). Neither Tyr promoter activity nor mRNA levels of tyrosinase, Tyrp1, and DCT were altered by short-term treatment of FSK or altiratinib, arguing against the nonspecific effect of altiratinib on CRTC3/CREB-mediated MITF expression (Figure 8I, Fig. S16A). Altiratinib dose-dependently reduced melanin content (Fig. 8J, Figure S16B) but did not affect cell viability in NHM (Figure 8K). In epidermal melanocytes of the *ex vivo* human skin tissue, CRTC3 was expressed and UVB, a relevant cAMP signal, induced nuclear translocation of CRTC3 (Figure 8L). Also, altiratinib strongly attenuated UVB-induced melanin accumulation in *ex vivo* human skin explants (Figure 8M). Furthermore, UVB-stimulated elevation of MITF and tyrosinase expression in *ex vivo* human skin tissue was also suppressed by altiratinib (Figure 8N, O).

## Discussion

All cells and organs in the body are exposed to a variety of mutagenic agents during their lifetime, and the failure to maintain genomic stability results in various disorders, such as developmental defects, cancers, and premature aging. Although the DNA repair system protects the integrity of genetic information, skin, directly and constantly exposed to highly genotoxic UVB, has evolutionarily conserved skin pigmentation as an additional defense mechanism. In the skin, melanocytes possess the unique ability to synthesize melanin and transfer melanin-equipped melanosomes to adjacent keratinocytes, where they accumulate melanin caps above the keratinocyte nuclei to absorb UVB and clear the mutagenic photoproducts. Epidermal melanin protects against UVB-induced cutaneous DNA damage and tumorigenesis, especially in cancer-prone patients, among others with xeroderma-pigmentosum-complementation-group-C-deficient genetic background 20. Conversely, balanced epidermal melanogenesis is essential for human health and survival, and adequate UVB exposure is encouraged to maintain homeostasis, such as preventing rickets through vitamin D3 synthesis.

Recently, naturally occurring pigmentation-mutant mice have been used to investigate molecular mechanisms underlying melanin pigmentation. The cloning of *agouti signaling peptide (ASIP)* and *melanocortin receptor 1 (MC1R)* genes from *a (agouti)* and *e (extension)* loci and characterization of POMC-derived melanogenic peptide in MC1R signaling led to the recognition of the fundamental role of the cAMP signaling pathway in melanogenesis 21-24. Physiologically, UVB increases p53 levels in keratinocytes, leading to increased expression of SCF and POMC 25. Derived from POMC, αMSH/ACTH increases intracellular cAMP and protein kinase A (PKA) activity in melanocytes through MC1R. Then in melanocytes, upon phosphorylation, CREB, a ubiquitously expressed basic leucine zipper (bZip) transcription factor, recruits CBP/p300 and binds the cAMP-responsive element (CRE) motif of the regulatory regions of the target genes, including *MITF* 26. Reflecting the central role of the cAMP signaling pathway in melanogenesis, treatment with the PDE4 inhibitors, rolipram and Ro31-1724, alone or in combination with FSK, induced robust melanization 27 and mice deficient in PDE4d exhibited darker skin 28. In humans, patients with acrodysostosis carrying mutations in PRKAR1A that constitutively activates PKA, present with pigmented skin lesions 29. Also, pigmented schwannomas have been observed in Carney's complex patients 30.

Although these studies documented the significance of cAMP-CREB-MITF pathway in melanogenesis, *in vivo* genetic evidence confirming the key role of CREB-mediated transcription in this pathway is lacking. Systemic deletion of the bZip domain or exon 2 CREB knock-out mice resulted in either perinatal death or did not show a discernible phenotype 31-33. The current study is consistent with our previous studies, indicating that cytoplasmic and nuclear shuttling of CRTC3 could determine CREB activity and melanogenesis 16 , 34 without altering CREB phosphorylation status, and provides compelling *in vivo* genetic evidence that CRTC3/CREB plays a key role in melanogenesis. In contrast to CREB null mice, which did not show identifiable pigmentation phenotypes, deletion of CRTC3 resulted in pale skin and hair color, as determined by biochemical and pharmaceutical studies. These observations suggested that CREB function might be compensated by the splicing variant isoforms of CREB and other CREB family members, such as ATF1 and CREM 32, 35, whereas CRTC3 was irreplaceable for CREB-mediated MITF expression.

Although the expression of CRTC1 and CRTC2 was observed in cultured melanocytes and/or melanoma cell lines 16, 36, CRTC1 and CRTC2 null mice did not present any skin and coat color changes 5, 6 (and personal communication). Recently, CRTC1 has been reported as a target to improve hyperpigmentation. Since CRTC1 and CRTC3 likely share the upstream signaling regulatory mechanisms, depigmentation effects of the agents targeting CRTC1 might be due to the inhibition of CRTC3 activity* in vivo* 36. Previously, we and other researchers observed overlapping expression of CRTC2 and CRTC3, but independent phenotypes occurred in the liver and adipose tissue of CRTC2 and CRTC3-null mice 7, 8, suggesting the presence of other sites enabling CRTC2/CRTC3 to specifically regulate subset of CREB target gene expression. Although both CRTC2 and CRTC3 utilize CREB for the transcription of genes containing the CRE element, they may have their own target specificity, conferred by cis-elements in each gene- or cell-specific transcription cofactors favoring CRTC2 or CRTC3. Thus, PEPCK and G6Pase in the liver while MITF in melanocytes may be preferentially regulated by CRTC2 and CRTC3, respectively. Also, the expression levels of CRTC2 and CRTC3 in cultured cells versus *in vivo* might be different. For example, we noticed that CRTC3 expression level was minimal and difficult to detect in the liver but was significantly elevated in primary cultured hepatocytes. Similarly, the expression of CRTC2 or CRTC1 in melanocytes *in vivo* might be much less than in cultured melanocytes. These hypotheses need to be confirmed in the future.

MITF is one of the most complex genes involved in almost all aspects of melanocyte biology, including development (lineage differentiation), survival, proliferation, and melanogenesis (maturation) 37, 38. Among multiple MITF isoforms, melanocytes express MITF‐A and MITF‐M. MITF‐A knock-out mice showed only subtle changes in hair and skin pigmentation, but the targeted deletion of the MITF‐M isoform resulted in the lack of neural crest derived-melanocytes and loss of melanocytes in the epidermis, hair follicle, iris, and choroid 39. Because CREB is known to regulate MITF-M expression specifically, we evaluated melanogenesis and melanocyte development in CRTC3-null mice. Melanoblasts in the neural crest are found on embryonic day 10.5 (E10.5). Following their migration and proliferation, epidermal melanocyte development is established by E14, and the SOX10+ melanocytes found in the epidermis are fully developed pigment-producing cells 40. MITF expression during melanocyte development is primarily dependent on SOX10 and PAX3, and a certain level of MITF expression established by SOX10 appears to be sufficient for melanocyte proliferation and migration 41. In agreement with the published reports 41, CRTC3-null mice did not show abnormalities in eye development (eye size and color) and the number of melanocyte marker SOX10+ epidermal melanocytes was not reduced, suggesting that CRTC3 deficiency did not affect the development or survival of melanocyte lineage. Our data suggested that CRTC3 directly targeted and quantitatively regulated MITF; the light hair and skin color phenotypes in CRTC3-null mice ultimately resulted from decreased Tyr expression following the downregulation of MITF.

Like CRTC3-null mice, transgenic mice that express shRNAi targeting Tyr with approximately 60% reduced Tyr expression displayed light hair color with reduced melanin content. These mutant mice exhibited fewer stage IV melanosomes, suggesting that impaired melanosome maturation in CRTC3-null mice was partially caused by reduced tyrosinase expression 42. Our KRT14‐SCF: CRTC3-null cross model provided further evidence, showing that the SOX10+ epidermal melanocyte population was not diminished compared to the control KRT14‐SCF mice, whereas KRT14‐SCF: CRTC3-null mice exhibited a substantial reduction in melanin accumulation in the epidermis compared to the KRT14-SCF mouse skin.

A recent study describing CRTC3-null mice 43 and CRTC3 function in melanocyte differentiation and melanoma oncogenesis reported coat color phenotypes similar to those observed in our study. However, in contrast to our study, downregulation of MITF, tyrosinase, and DCT expression in CRTC3-null mice was not observed and instead OCA2 was proposed as a CRTC3 target. The discrepancy could be due to the use of hair-bearing dorsal skin tissue. The hair follicular melanogenesis regulation is different from epidermal melanogenesis, because the melanogenic gene expression of hair follicular melanocytes mainly depends on the hair cycle 44, 45 and may override the reduction of melanogenic genes in the epidermis of neonatal CRTC3-null mice. In the C57BL6/J genetic background, epidermal melanocytes are found only in the ear, tail, and sole skin tissues 13. We excluded hair follicles to avoid complexity and analyzed epidermal melanogenesis in the tail or ear skin and observed a marked downregulation of the expression of melanogenic genes. We also attempted to identify cAMP/CRTC3 target genes responsible for CRTC3-null mice phenotypes. However, except for MITF, all other melanogenesis-associated genes that were downregulated in the CRTC3 knock-out melanocytes and the skin of CRTC3-null mice was not sensitive to FSK treatment. For example, we found well-conserved CRE sites in the promoter regions of DCT and OCA2; however, FSK-induced increase in DCT and OCA2 expression was gradual and not observed until 12 h after FSK treatment. Not all CREB target gene expression followed burst-attenuation kinetics upon FSK treatment; however, because both DCT and OCA2 contain an M-box and could be potentially regulated by MITF, it was difficult to determine whether their transcription was regulated by CRTC3 and/or MITF. Analysis of the FSK-stimulated gene pattern expression in MITF knock-down or knock-out melanocytes might provide more accurate information.

Collectively, we showed that the CREB co-activator CRTC3 directly targeted and quantitatively influenced MITF to regulate the degree of melanin synthesis. Our data indicated that melanocyte CRTC3 accounted for the hair and skin color phenotype of CRTC3-null mice. However, the intrinsic limitations of the systemic knockout model did not completely exclude the involvement of other cells and tissues *in vivo*. Generation of melanocyte-specific CRTC3-null mice and confirming whether melanocyte-specific conditional null mice phenocopy systemic CRTC3-null mice would provide more accurate information. So far, 243 genes and 325 pigment-related genes have been identified from mouse studies. Among these, 51 human pigment genes have been characterized 46. However, even after several decades of investigation, epidermal melanogenesis is still poorly understood. MITF is undeniably the central transcription factor in melanocytes. Therefore, a reversible, tunable, and highly sophisticated system that includes signaling pathways, nuclear shuttling, and more precise regulatory mechanisms, is essential to elucidate MITF regulation in melanocytes, involving an upstream regulator (for example, CRTC3) is needed 47.

Previously, the development of vitiligo or contact leukoderma by rhododendrol and other depigmenting agents, detrimental to skin homeostasis leading to melanocyte death or toxicity, has been reported. Melanocyte loss and irreversible depigmentation resulted in treatment-resistant vitiligo 48-50. Despite the multidimensional action of MITF, melanocyte lineage development or survival was not affected in CRTC3-null mice. Our results are especially relevant for the selective regulation of melanin synthesis by the CRTC3/MITF pathway. Furthermore, by screening for CRTC3/CREB activity, we identified suitable candidate agents. We found that altiratinib could downregulate cAMP- or UVB induced melanogenesis without affecting human melanocyte viability. Altiratinib is a multi-receptor kinase inhibitor that selectively targets HGF-c-Met, VEGF-VEGFR2, NGF-TRK (NGFR) signaling pathways 51. Since HGF and NGF are recognized melanogenic paracrine factors associated with solar lentigo and melasma pathogenesis, common hyperpigmentary skin disorders 52-55, the anti-melanogenic activity of altiratinib could potentially be mediated by interfering with these signaling pathways. While our data suggested crosstalk between these signaling pathways and CRTC3, the mechanism of altiratinib underlying CRTC3 phosphorylation and inhibition of CREB/CRTC3 activity remains to be clarified. In this context, because ERKs and CDKs can activate CRTC3/CREB-mediated transcriptional activity via recruitment of PP2A to CRTC3 56, the inhibitory activity of altiratinib against ERKs could be one possibility 57. Alternatively, given the multifaceted action of altiratinib, CRTC3 phosphorylation induced by altiratinib could be due to the combined inhibition of various signaling pathways. Further studies are needed to better understand the molecular mechanisms underlying CRTC3 phosphorylation and crosstalk between signaling pathways, extending the discovery of novel targets for the regulation of CRTC3 activity and melanogenesis.

In conclusion, understanding the detailed molecular mechanisms of epidermal pigmentation by CRTC3 modulation could lead to the development of new strategies to protect against UVB-induced skin carcinogenesis and treat hyper- or hypo-pigmentation skin disorders. Since this strategy appeared to work well in normal human melanocytes and human skin tissue, it may provide therapeutic benefits for pigmentation disorders without affecting melanocyte integrity, opening a clinical application possibility of safe and effective use.

## Supplementary Material

Supplementary figures and table.

## Abbreviations

CRTC3: CREB-regulated transcriptional co-activator 3
DOPA: 3,4-dihydroxy-L-phenyl-alanine
DHICA: 5,6-dihydroxyindole-2-carboxylic acid
DHI: 5,6-dihydroxyindole
TRP-1: Tyrosinase related protein-1
DCT (TRP-2): Tyrosinase related protein-2
MITF: Microphthalmia-associated transcription factor
cAMP: cyclic adenosine monophosphate
PKA: protein kinase A
CREB: cAMP response element binding protein
MAPK: Mitogen-activated protein kinase
ERK: Extracellular signal-regulated kinase
MSH: melanocyte stimulating hormone
ACTH: adrenocorticotropic hormone
POMC: pro-opiomelanocortin
