# Supplementary material for: CRTC3, a sensor and key regulator for melanogenesis, as a tunable therapeutic target for pigmentary disorders
Source: Theranostics. 2021 Oct 17;11(20):9918–36. doi: 10.7150/thno.66378 (PMC8581419; doi:10.7150/thno.66378)
Supplement: Supplementary file 1 — Supplementary figures and table. [file thnov11p9918s1.pdf]

Supplementary materials

Supplementary Table 1. List of primers used for qRT-PCR (F: Forward, R: Reverse)

**Name**	**Forward (3' → 5')**	**Reverse (5' → 3')**
mL32	TCTGGTGAAGCCCAAGATCG	CCTCTGGGTTTCCGCCAGTT
mCRTC3	GAAGTTCAGTGAGAAGATCGC	CCCCGTGGTACTGGGTAAG
mNR4A2	AGTCTGATCAGTGCCCTCGT	GATCTCCATAGAGCCGGTCA
mCREB	CCAGTCTCCACAAGTCCAAACA	GGCACTGTTAGAGTGGTGGTATG
mERK1	CCTGCTGGACCGGATGTTA	TGAGCCAGCCTTCCTCTAC
mERK2	GGAGCAGTATTATGACCCAAGTGA	TCGTCCACTCCATGTCAAACT
mMLANA	GACGAAGTGGATACAGAACCTTG	CTCTTGAGAAGACAGTCGGCTG
mMITF-M	GGGATGCCTTGTTTATGGTG	CACCGCAGACCACTTAGTCC
mTyrp1	CCCCTAGCCTATATCTCCCT	TACCATCGTGGGGATAATGG
mDCT	CTTTGCAACCGGGAAGAACG	CCGACTAATCAGCGTTGGGT
mTyr	TTATGCGATGGAACACCTGA	GAGCGGTATGAAAGGAACCA
mPmel	CAAGTTCCCCTGGACTGTGT	GTGCTACCATGTGGCATTTG
mSOX10	CGGACGATGACAAGTTCCCC	GTGAGGGTACTGGTCGGCT
mSLC24A5	AGAGCACGGATGGAGGTATCGT	GCAACATCCTGCGACAGTCCAA
mSLC45A2	ACACAGAGCAGCCAGTACAGGA	CAATCAGGTGGCTGACGCAAAG
mOCA2	ATAGTGAGCAGGGAGGCTGT	ACTGATGGGCCAGCAAAAGA
mSCF	TCCGAAGAGGCCAGAAACTA	TCCCTTTCTCGGGACCTAAT
mET1	ACTTCTGCCACCTGGACATC	GGTGAGCGCACTGACATCTA
mBFGF	AAGCGGCTCTACTGCAAGAACG	CCTTGATAGACACAACTCCTCTC
mPOMC	AAGTGGAGATTCAACACCATTCTTAA	GTCCAGAGCTGAGACACCCTTAC
hGAPDH	[illegible]CATCTTCCAGGAG	GTTGTCATGGATGACCTTGGC
hCRTC3	GCACCAGCCTGTTCAAAGAC	TCTGCAGCTCCTCTTCCAGT
hMITF-M	TCTACCGTCTCTCACTGGATTGG	GCTTTACCTGCTGCCGTTGG
hTyrp1	CCCCTAGCCTATATCTCCCTTTT	TACCATCGTGGGGATAATGGC
hDCT	TGTGCAAGATTGCCTGTCTC	GTTGCTCTGCGGTTAGGAAG
hTyr	TCAGCACCCCACAAATCCTAA	AATCGGCTACAGACAATCTGC
hPmel	GAAGACCTGGGGGCCAATACT	TGAAGGCTGAGCTGGAATGA
hSOX10	ATGAACGCCTTCATGGTGTGGG	CGCTTGTCACTTTCGTTCAGCAG
hSLC24A5	AGCGCAGAGATGGAGGCATCAT	TGTGCCTGCAACATCCTGAGAC
hSLC45A2	CTTTGCATCAGCCACCTCATTGG	TCCAACCTCGACTCCTCTTTCG

hOCA2	AGGAGAAGCGAGCACTCAGTGA	CACCTGGGTTTCTACACTTCCG
hSCF	TGGTGGCAAATCTTCCCAAAAAG	CAATGACTTGGCAAAACATCCA
hET1	AAGGCAACAGACGCTGAAAAT	CGACCTGGTTTGTCTTAGGTG
hBFGF	AGCGGCTGTACTGCAAAAACGG	CCTTTGATAGACACAACTCCTCTC
hPOMC	CTGGAGAGCAGCCAGTGTCAG	AGAGGCTGCTCGTCGCCATTTC

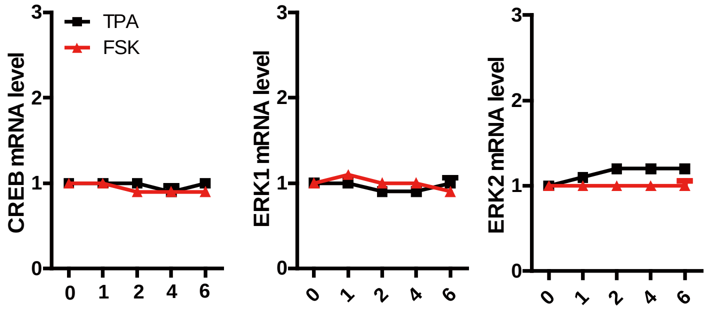

Fig. S1.

The mRNA levels of CREB, ERK1, and ERK2 in Mel-Ab cells treated with FSK or TPA.

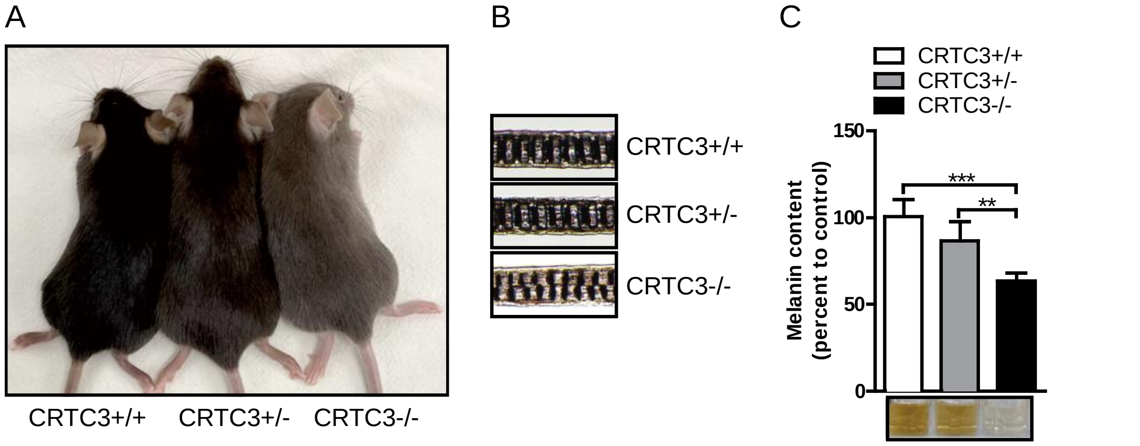

Fig. S2.

Lighter coat color and hypopigmentation of skin in CRTC3 null mice. (A) The comparison of hair color and (B) hair structure of CTRC3 wild type CTRL, CRTC3 heterozygotes and null mice (C) Quantification of melanin content of dorsal hair from CTRL, CRTC3 hetero and null mice (n = 3 per group, 2 month old).

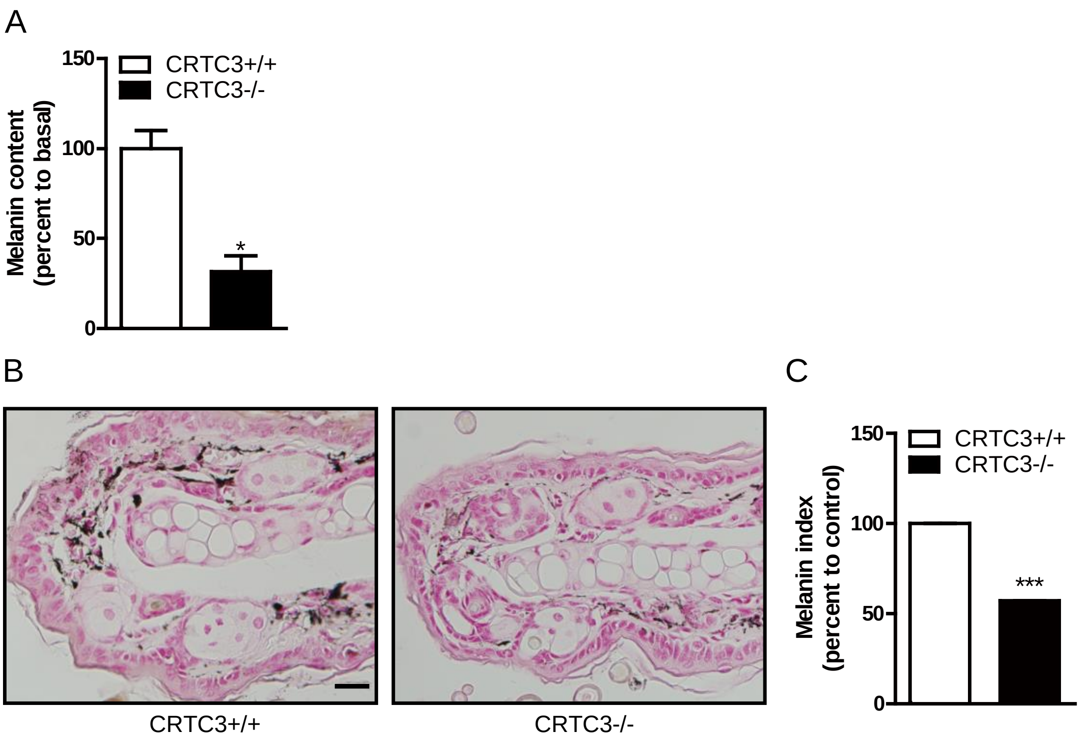

Fig. S3.

(A) Quantification of melanin content in tail skin from CTRL and CRTC3 null mice (B) Histology of ear skin and (C) melanin index displayed as percent to control from CTRL and CRTC3 null mice (Bar = 20 μm).

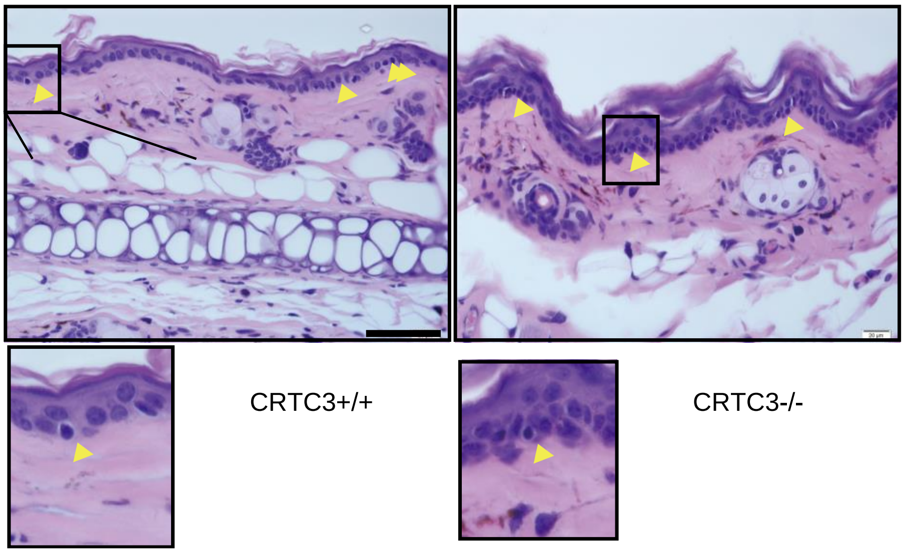

Fig. S4.

Comparison of epidermal melanocytes (yellow arrow) in ear skin of CTRL and CRTC3 null mice (Bar = 50 μm).

Fig. S5

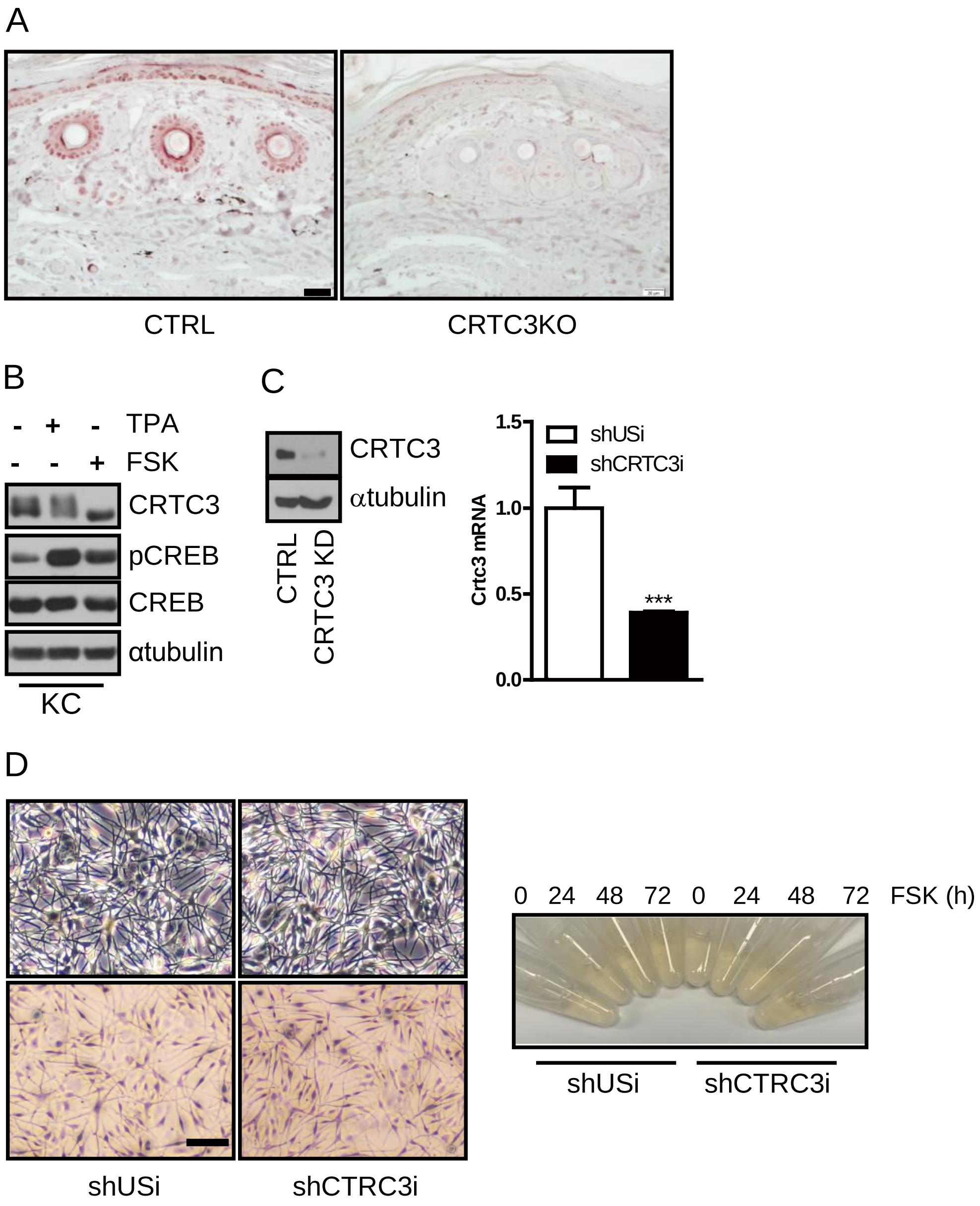

Fig. S5.

(A) Immunohistochemistry using CRTC3 antibodies in the tail skin of CTRL and CRTC3 null mice (Bar = 50 μm). (B) Expression and response of CRTC3 and CREB to FSK and TPA stimulation in normal human keratinocytes. (C) Protein and mRNA levels of CRTC3 in control and CRTC3KD keratinocytes. (D) Microscopic images of normal human melanocytes (NHM) co-cultured with either control HaCaT keratinocytes or CRTC3KD keratinocytes after 72 h with/without FSK treatment (left panel) and cell lysates showing melanin content (right panel) (Bar = 100 μm).

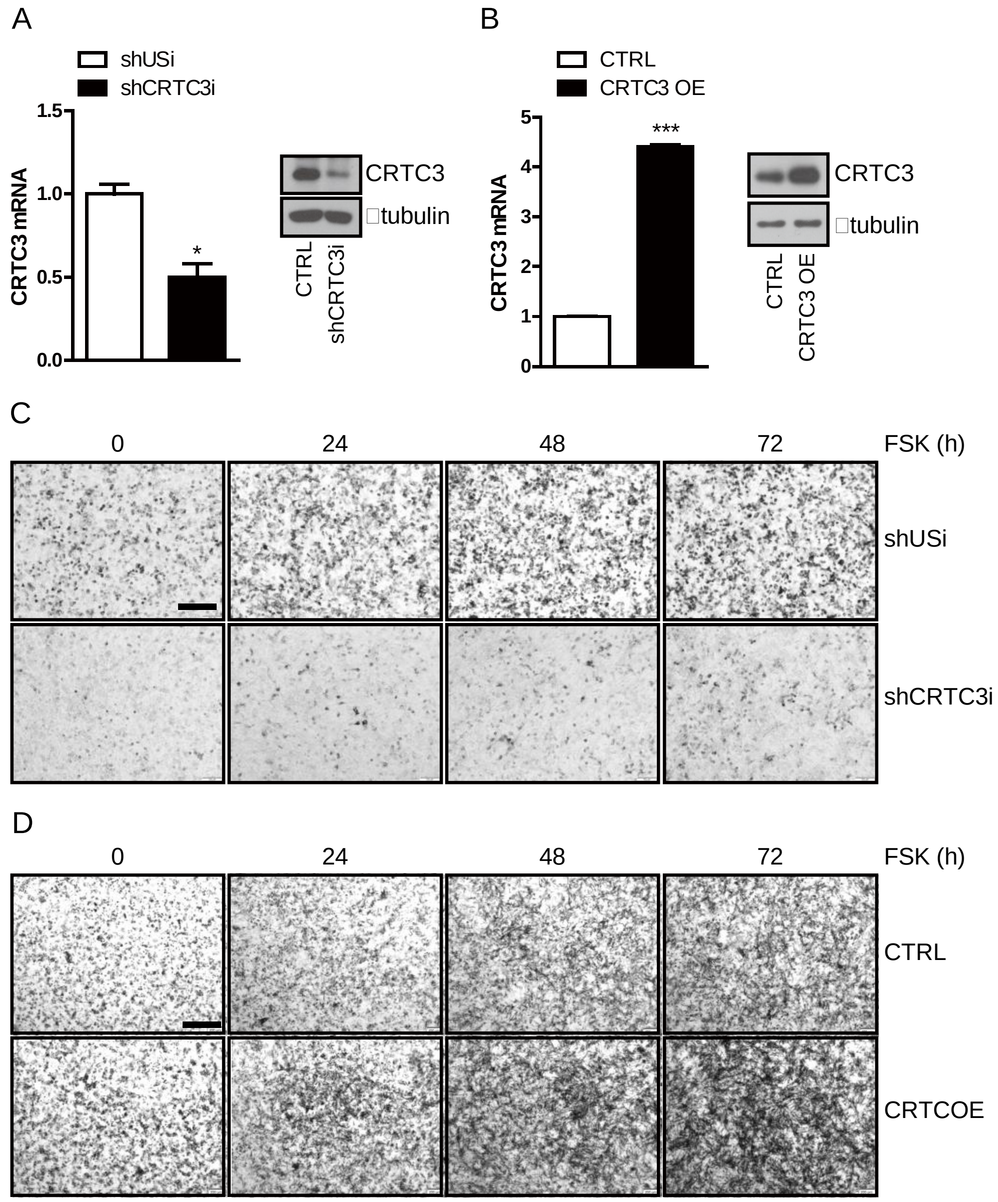

Fig. S6.

(A) mRNA and protein levels in control and CRTC3KD Mel-Ab cells. (B) mRNA and protein levels in control and CRTC3 overexpressing (OE) Mel-Ab cells. Microscopic images of (C) control and CRTC3KD Mel-Ab cells and (D) control and CRTC3OE Mel-Ab cells at 0, 24, 48, 72 h after FSK treatment (Bar = 500 μm).

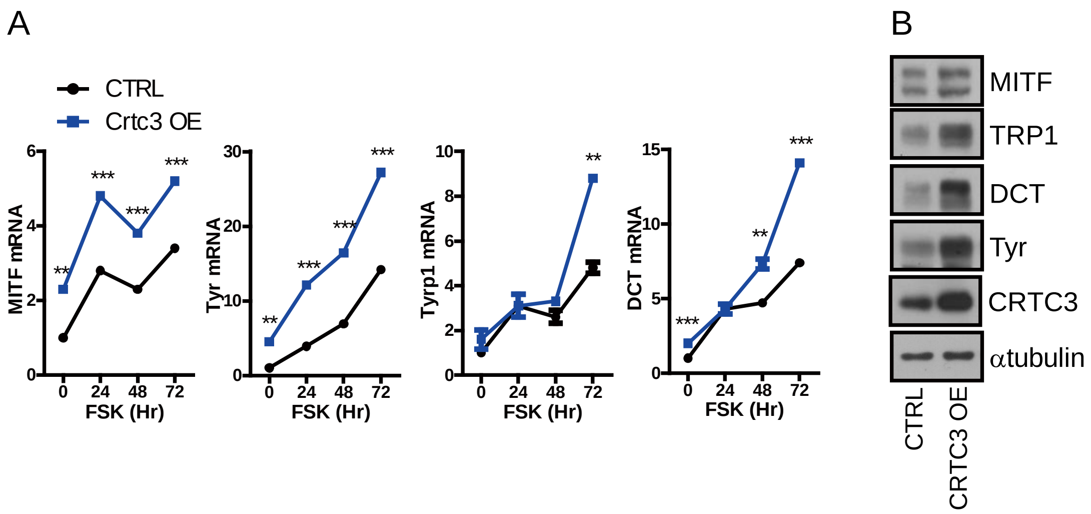

Fig. S7.

(A) mRNA and (B) protein levels in control and CRTC3 overexpressing (OE) Mel-Ab cells at 0, 24, 48, 72 h after FSK treatment.

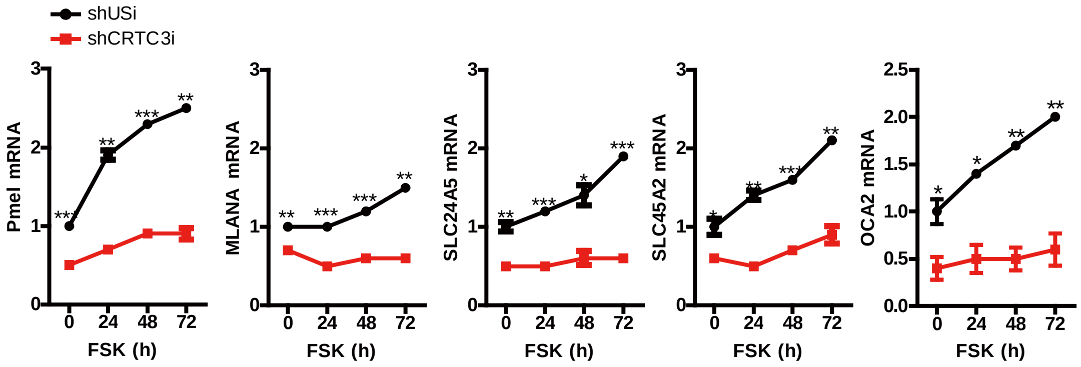

Fig. S8.

mRNA level of melanogenesis related genes in control and CRTC3KD Mel-Ab cells at 0, 24, 48, 72 h after FSK treatment.

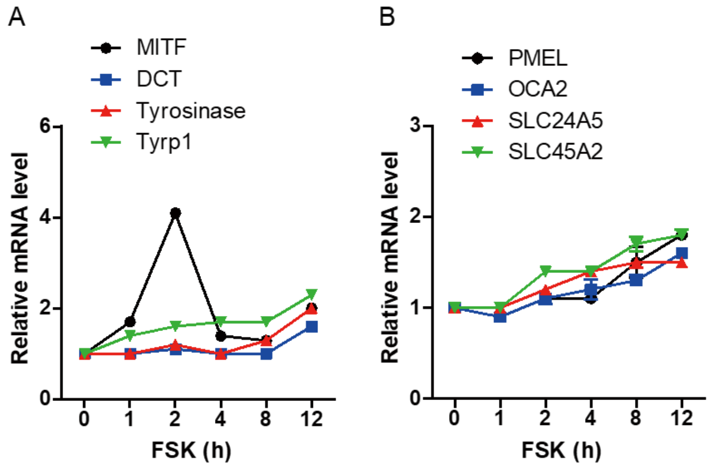

Fig. S9.
(A-B) mRNA level of melanogenesis-related genes in B16F10 melanoma cells within 12 h after FSK treatment.

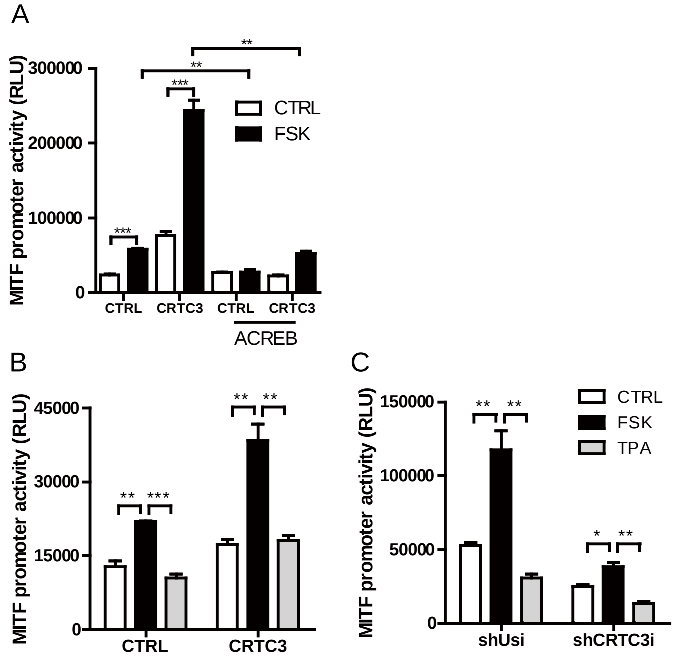

Fig. S10.
(A) MITF promoter activity by FSK treatment, CRTC3 and/or ACREB overexpression in B16F10 melanoma cells. Comparison of MITF promoter (B) in control and CRTC3 overexpressing B16F10 melanoma cells and (C) in control and CRTC3KD B16F10 melanoma cells.

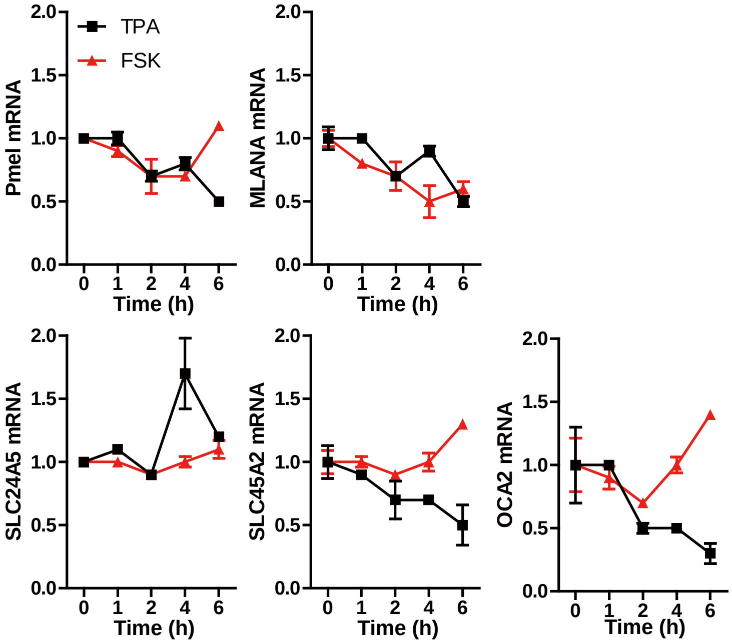

Fig. S11.

mRNA level of melanogenesis-related genes in Mel-Ab cells within 6 h after FSK or TPA treatment.

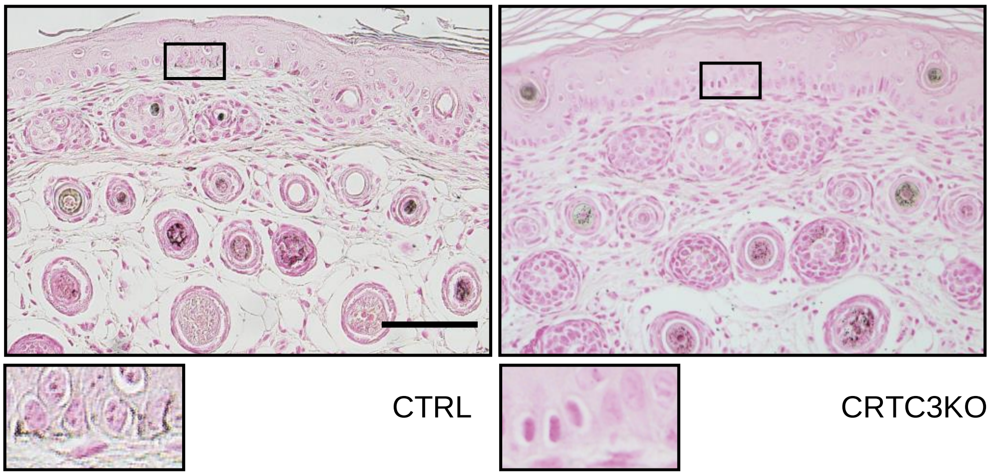

Fig. S12.

Microscopic images of Fontana-Masson stained tail skin sections from neonatal CTRL and CRTC3 null mice (Bar = 50 μm).

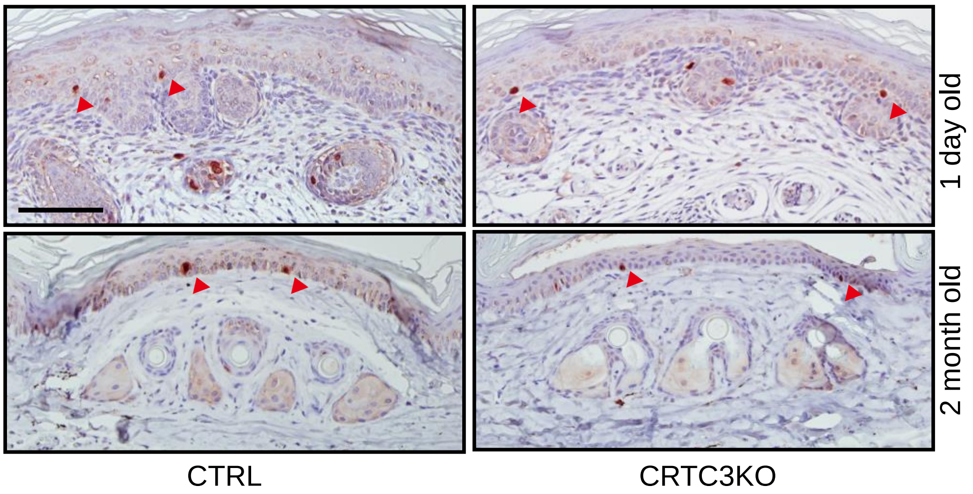

Fig. S13.

Immunohistochemistry using Ki67 antibody in the tail skin of neonatal CTRL and CRTC3 null mice (Bar = 50 μm).

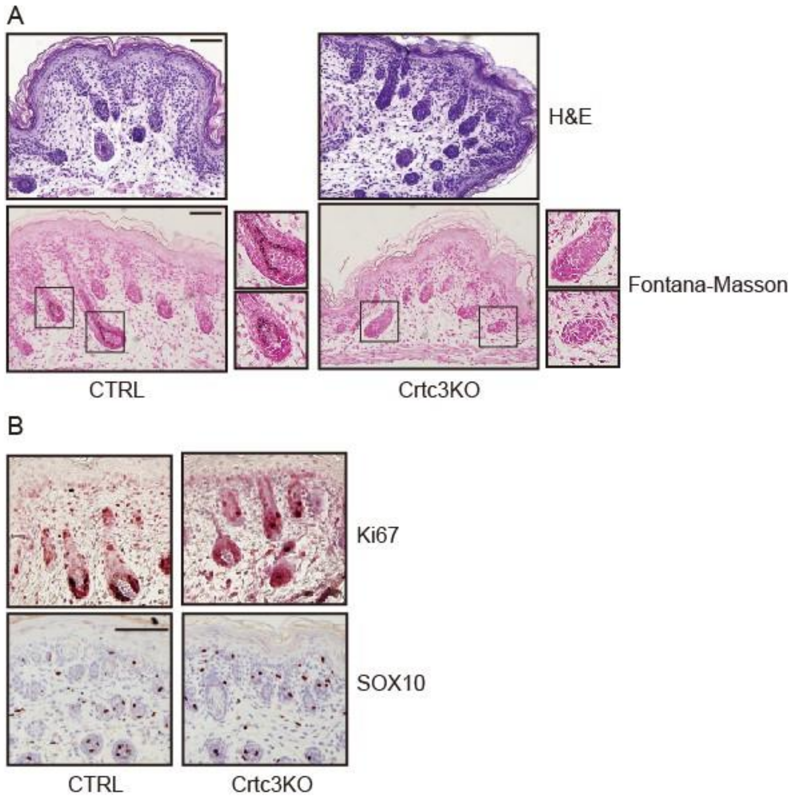

Fig. S14.

(A) H&E (upper panels) and Fontana-Masson (low panels) stained dorsal skin sections from neonatal CTRL and CRTC3 null mice (Bar = 50 μm). (B) Immunohistochemistry using Ki67 (upper panels) and SOX10 (downer panels) antibodies in the dorsal skin of neonatal CTRL and CRTC3 null mice (Bar = 50 μm).

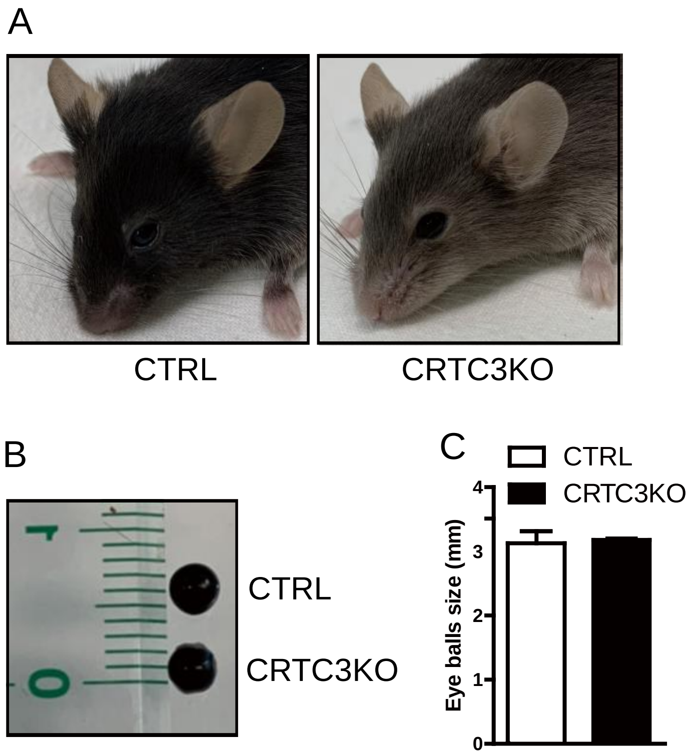

Fig. S15.
Closed-up pictures of (A) eyes and (B) dissected eyeballs of CTRL and CRTC3 null mice. (C) eyeball size (n=3 for each group).

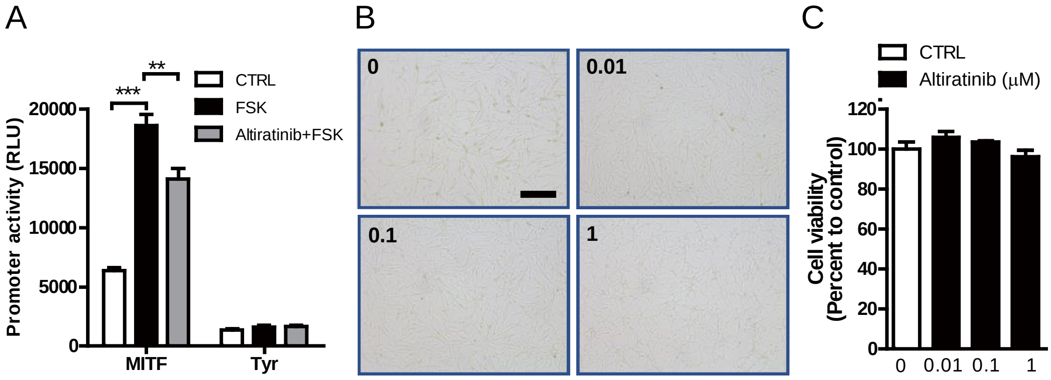

Fig. S16.
The effect of FSK and altiratinib on MITF and tyrosinase promoter activity. (B) Microscopic images of primary human melanocytes at 72 h after 0, 0.01, 0.1, and 1μM of altiratinib treatment (Bar = 1000), (C) The effect of altiratinib (0.01-1 μM) on cell viability of HaCat human keratinocytes cell line as assed by MTT assay.
